# Maize plant architecture trait QTL mapping and candidate gene identification based on multiple environments and double populations

**DOI:** 10.1186/s12870-022-03470-7

**Published:** 2022-03-11

**Authors:** Jianbo Fei, Jianyu Lu, Qingping Jiang, Zhibo Liu, Dan Yao, Jing Qu, Siyan Liu, Shuyan Guan, Yiyong Ma

**Affiliations:** 1 College of Bioscience, Jilin Agricultural University, Changchun, 130118 China; 2 College of Agriculture, Jilin Agricultural University, Changchun, 130118 China

**Keywords:** Quantitative trait loci (*QTL*), Maize, Plant height, Ear height, Leaf angle above the primary ear, Internode length above the primary ear

## Abstract

**Background:**

The plant architecture traits of maize determine the yield. Plant height, ear position, leaf angle above the primary ear and internode length above the primary ear together determine the canopy structure and photosynthetic efficiency of maize and at the same time affect lodging and disease resistance. A flat and tall plant architecture confers an obvious advantage in the yield of a single plant but is not conducive to dense planting and results in high rates of lodging; thus, it has been gradually eliminated in production. Although using plants that are too compact, short and density tolerant can increase the yield per unit area to a certain extent, the photosynthetic efficiency of such plants is low, ultimately limiting yield increases. Genetic mapping is an effective method for the improvement of plant architecture to identify candidate genes for regulating plant architecture traits.

**Results:**

To find the best balance between the yield per plant and the yield per unit area of maize, in this study, the F2:3 pedigree population and a RIL population with the same male parent were used to identify *QTL* for plant height (PH), ear height (EH), leaf angle and internode length above the primary ear (LAE and ILE) in Changchun and Gongzhuling for 5 consecutive years (2016–2020). A total of 11, 13, 23 and 13 *QTL* were identified for PH, EH, LAE, and ILE, respectively. A pleiotropic consistent *QTL* for PH overlapped with that for EH on chromosome 3, with a phenotypic variation explanation rate from 6.809% to 21.96%. In addition, there were major consistent *QTL* for LAE and ILE, and the maximum phenotypic contribution rates were 24.226% and 30.748%, respectively. Three candidate genes were mined from the three consistent QTL regions and were involved in the gibberellin-activated signal pathway, brassinolide signal transduction pathway and auxin-activated signal pathway, respectively. Analysis of the expression levels of the three genes showed that they were actively expressed during the jointing stage of vigorous maize growth.

**Conclusions:**

In this study, three consistent major *QTL* related to plant type traits were identified and three candidate genes were screened. These results lay a foundation for the cloning of related functional genes and marker-assisted breeding of related functional genes.

**Supplementary Information:**

The online version contains supplementary material available at 10.1186/s12870-022-03470-7.

## Background

Maize is the most important food crop and industrial raw material in the world. Increasing maize production is critical for ensuring food security and industrial development [1]. In the past half century, the increase in maize production was mainly the result of increased planting density [2]. Since the 1930s, the maize planting density in the United States has increased from 30,000 plants/hm^2^ to the current 8.55 ~ 109,500 plants/hm^2^, and the yield has increased from 1,500 kg/hm^2^ to the current 11,000 kg/hm^2^, a 7.3-fold increase. In China, the planting density and yield of maize are also increasing, from less than 30,000 plants/hm^2^ in 1949 to the current 52,000–60,000 plants/hm^2^, with the yield of maize kernels increasing from 960 kg/hm^2^ to the current 6,316 kg/hm^2^, while the yield per unit area increased by nearly 6.6 times (National Statistical Yearbook, 2019, http://www.gov.cn/shuju/2019-12/07/content_5459250.htm). Although the biomass and yield per plant decreased with the increase in planting density, the biomass and yield per unit area increased. However, this relationship is not static [3–6]. When the density is excessively high, plants are stressed, eventually leading to a decline in yield per unit area. Appropriate plant architecture is a crucial prerequisite for high-density maize production [7–11].

**Fig. 1:**
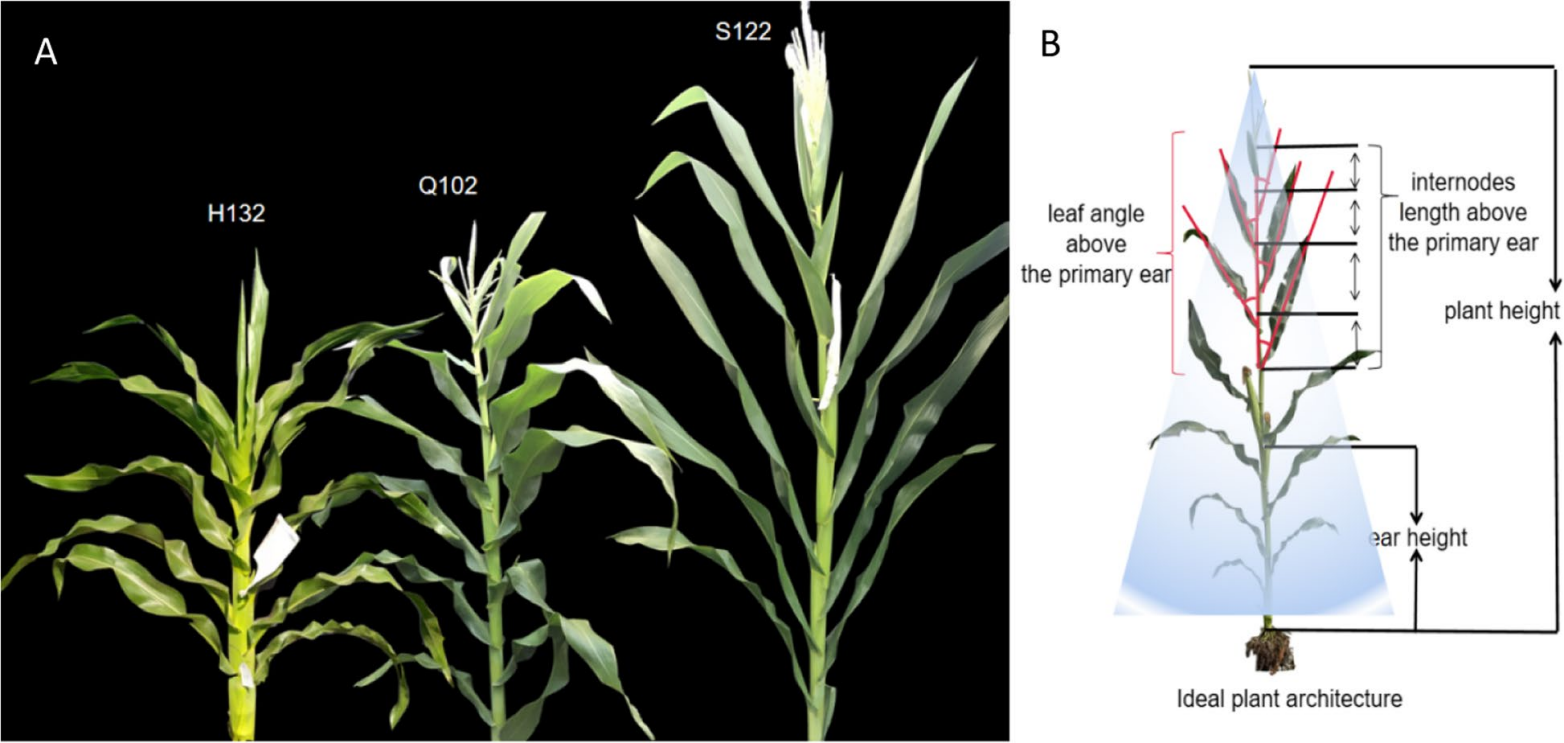
Maize plant architecture traits. **A** Parent plant architecture of two populations (F_2:3_ and RIL). **B** Schematic diagram of the ideal plant architecture and measurement positions of plant architecture traits

Maize plant architecture refers to the spatial distribution of maize plants, including plant height (PH), ear height (EH), leaf angle, and internode length [12, 13]. PH and EH are important factors that affect the architecture of maize plants [14]. On the one hand, an optimal PH and EH can increase planting density, maximize the utilization of fertilizers and water, and promote effective photosynthesis. On the other hand, it can also improve the lodging resistance of plants and is more conducive to mechanical harvesting [15]. Although there have been many studies on the leaf angle in recent years, it lacks pertinence [16]. For an ideal plant type, the plant should have a smaller leaf angle above the primary ear (LAE) and a larger leaf angle at the primary ear position and below, giving the entire plant has a tower shape (Fig. 1B) [17]. Such a structure enables high light transmittance between the canopy layers, which minimizes shielding between the canopy layers, while ensuring the photosynthetic efficiency of the leaves at and under the primary ear position [18]. At the same time, the internode length above the primary ear (ILE) can change the distance between the leaves above the primary ear, and, in combination with a reasonable LAE, it can improve the canopy structure of maize [19]. The LAE and ILE directly affect both light distribution in the canopy and the utilization of light energy of the population, ultimately leading to yield changes [20]. The photosynthates used for grain filling mainly come from leaves above the primary ear. Plants with a smaller LAE and a longer ILE have potentially greater photosynthetic potential under dense planting conditions [21]. At the same time, improving the canopy structure of plants is also conducive to ventilation under dense planting conditions, reducing the humidity under the canopy, and improving the resistance of maize to various leaf spot diseases [22]. Breeding new hybrids by optimizing plant architecture is one of the most effective methods to increase the yield per unit area. Analyzing the genetic mechanism of maize plant architecture can provide crucial clues for identifying key genes or *QTL* in molecular breeding.

The detection of *QTL* is one of the most effective ways of identifying the genetic mechanisms underlying plant architecture traits. Our predecessors have carried out relevant work on the plant architecture traits of maize [23–27]. The MaizeGDB (http://www.maizegdb.org/) database has published 283 *QTL* related to plant height and 46 *QTL* related to ear height in maize. Most of the *QTL* cannot meet the needs of in-depth research due to early technical limitations and low positioning accuracy. However, with technological updates, the use of SNPs as markers has greatly increased the density of genetic maps, and the accuracy of positioning has also been greatly improved. *QTL* analysis based on high-density linkage maps will provide a basic understanding of the genetic structure of quantitative traits, thereby linking specific genetic loci with the biological mechanisms underlying ideal phenotypes [28]. The location and description of *QTL* help breeders understand the genomic regions related to complex traits and their contribution to the phenotype [29]. Wang et al. used SNP molecular markers to map plant heights of RIL populations for *QTL* mapping. The *QTL* were distributed on chromosomes 1, 3, 4, 5, 7, 8, and 10 [30]. In 2020, Ertiro et al. used DH populations to map yield-related traits of maize under low nitrogen stress and mapped 25 *QTL* for PH and 30 *QTL* for EH [29]. Since *QTL* are susceptible to environmental interference, it is difficult to find consistent *QTL* in studies of different genetic backgrounds and environments, which also makes it difficult for existing research to be applied to molecular-assisted selection [29]. In this study, S122 was used as the male parent, and H132 and Q102 were used as the two female parents to construct a F2:3 family population with 217 lines and a RIL population with 209 lines, respectively (Fig. 1A). Two genetic maps were constructed using SSR and SNP markers, and *QTL* were identified and mapped for PH, EH, LAE and ILE in the two populations in two districts (Changchun and Gongzhulign) for 5 consecutive years (2016–2020). The purpose was to analyze the genetic structure of the populations, find consistent *QTL* closely related to the target traits in different genetic backgrounds and environments, and mine candidate genes in the *QTL* intervals to verify the expression levels and functions of the candidate genes.

## Results

### Construction of a genetic linkage map

**Fig. 2:**
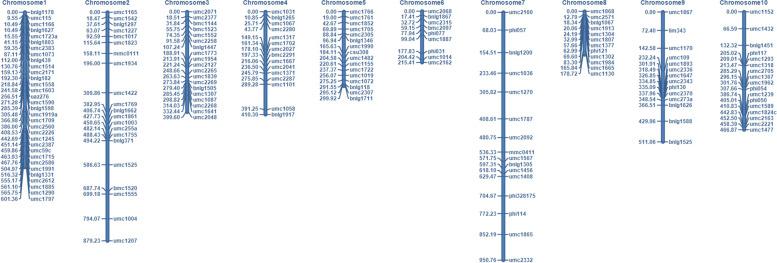
Genetic map of the F2:3 family population using SSR markers. The genetic locations are indicated on the left side of the chromosome, and the markers are indicated on the right side

The F2:3 population was screened for polymorphic primers, from 800 pairs of SSR primers to 171 pairs of polymorphic primers. The F2:3 population was genotyped with 171 pairs of SSR markers. Those consistent with the paternal genotype were recorded as 2, those consistent with the maternal genotype were recorded as 0, those consistent with the F1 genotype were recorded as 1, and the deletion was recorded as -1. Using ICIMapping 4.2 software(http://www.isbreeding.net), a genetic map covering 10 maize chromosomes with a total length of 4734.51 cM was drawn (Fig. 2). The average distance between adjacent markers in the map was 27.69 cM, the minimum distance was 0.24 cM, and the maximum distance was 113.86 cM. Comparing the genetic order of the genetic map markers with the physical sequence of IBM2008, the order was basically the same, indicating that the genetic map was accurate.

**Fig. 3:**
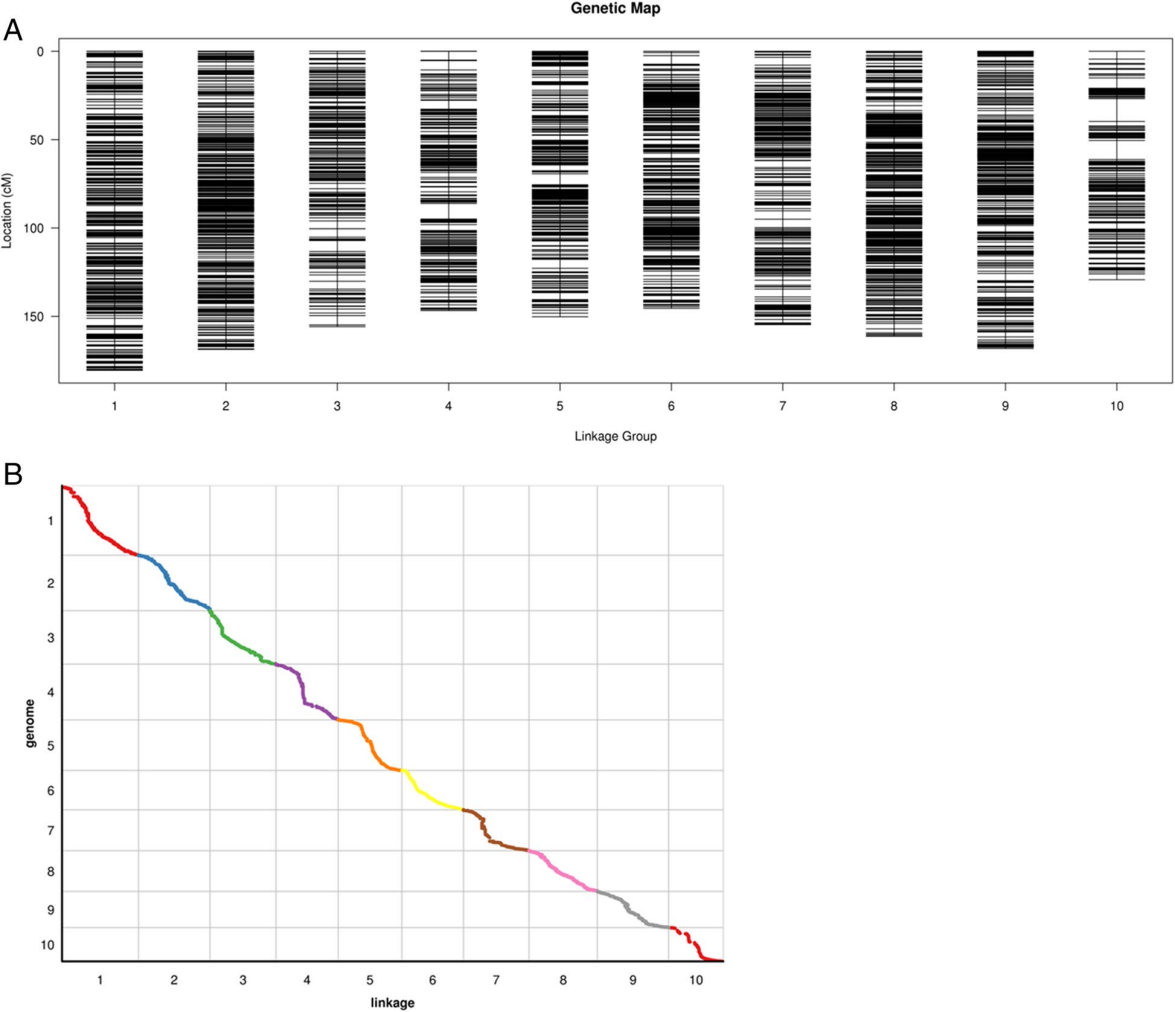
**A** High density genetic map of the RIL population using SNP markers. The markers are indicated by black bars. The x-axis represents 10 linkage groups, and the y-axis represents the genetic distance. **B** Genetic map and genome collinearity map. Both the x-axis and y-axis represent linkage group

The RIL population and parental genome were sequenced to obtain 19,624,498 SNP markers. Comparing the parental and progeny SNPs, 7,749,049 SNPs were successfully typed, filtering out the SNPs with more deletions and partial segregation in the progeny, and finally 5,130 SNP markers were identified that can be used for mapping. Taking the linkage group as a unit, HighMap [31] software was used to analyze the linear arrangement of the markers in the linkage group, and the genetic distance between adjacent markers was estimated. Finally a genetic map with a total length of 1,560.80 cM was obtained, and the average distance between adjacent markers was 0.308 cM (Fig. 3A). In this experiment, the sources of monomers in all linkage groups of each individual were counted, and possible double exchange sites were searched. There are two reasons for the occurrence of double exchange sites: (1) hot spots of genomic recombination and (2) typing errors caused by sequencing. In a linkage group, a higher proportion of double commutation indicates some problems in the typing or ordering of the atlas, usually controlled by a double commutation below 3%. The monomer origin assessment is shown in Figure S1, where the origin of larger segments in each individual remains consistent, indicating a high quality genetic map. The genetic map is essentially a multi-point recombination analysis. The closer the distance between markers, the smaller the recombination rate. Analyzing the reorganization relationship between the marker and the surrounding markers, we can find the markers with potential arrangement problems. A heat map of the linkage relationships of the linkage groups is shown in Figure S3 below. It can be seen from the heat map that the linkage relationship between adjacent markers on each linkage group in this project was very strong. As the distance increased, the linkage relationship between the markers gradually weakened, indicating that the marker order was correct. Through collinearity analysis, the Spearman coefficients between the genetic position and physical position of the SNP markers in each linkage group all exceeded 0.99 (Fig. 3B). The results showed that the order of most markers in each linkage group was consistent with the genomic results, and the calculation accuracy of the genetic recombination rate was high.

### Phenotypic analysis

**Fig. 4:**
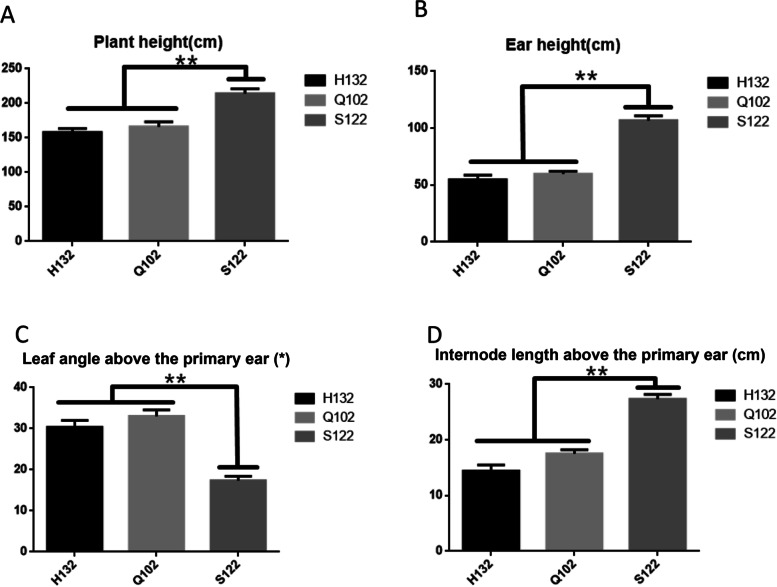
Inter-parental phenotypic data analysis. **A**: Difference analysis of plant height data between parents. **B**: Difference analysis of ear height data between parents. **C**: Difference analysis of leaf angle above the primary ear data between parents. **D**: Difference analysis of internode length above the primary ear data between parents. The asterisks (*or **) represent the significant differences at *p* < 0.05 or *p* < 0.01, respectively

Analysis of the phenotypic data of the parents showed that PH, EH, LAE and ILE were not significantly different between the two female parents. The phenotypic values of PH, EH and ILE of the male parent S122 were significantly higher than those in the two female parents. Conversely, the phenotypic values of LAE in two female parents were significantly higher than those in S122 (Fig. 4). Significant differences in phenotypic traits between male and female parents lay a good foundation for accurate *QTL* mapping.

**Fig. 5:**
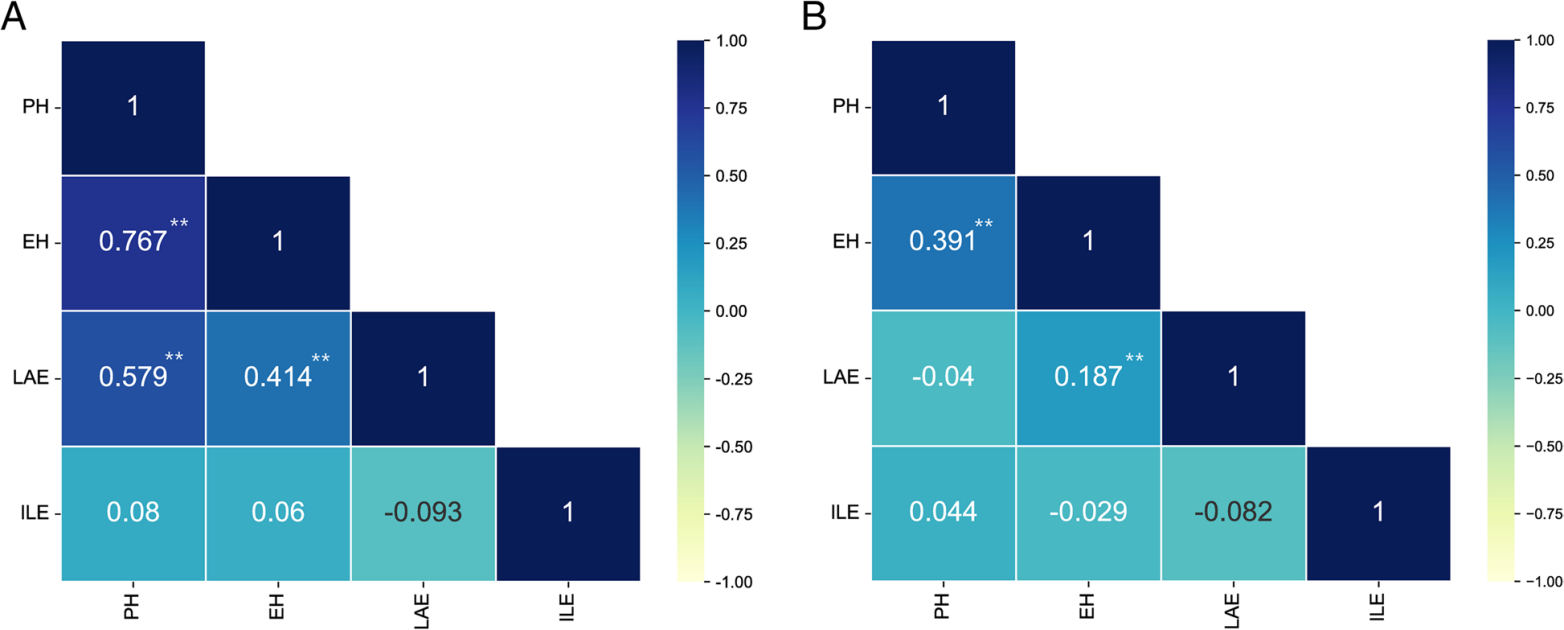
Pearson correlation coefficients among all plant architecture traits. **A**: Pearson correlation coefficient among plant architecture traits in the RIL Population. **B**: Pearson correlation coefficient among plant architecture traits in the F2:3 population. The asterisks (*or **) represent the significant differences at *p* < 0.05 or *p* < 0.01, respectively

The PH, EH, LAE and ILE of the populations generally showed a normal distribution (Figure S2). The average value, standard deviation, range, coefficient of variation skewness, kurtosis, and generalized heritability values of PH, EH, LAE and ILE are shown in Table 1. The range of the plant architecture data of the two populations was relatively large, and the coefficient of variation was small. This shows that the phenotypic difference was significant, and the degree of dispersion was low. Correlation analysis of the four phenotypic traits showed that the phenotypes of PH, EH, and LAE were significantly correlated in the RIL population (Fig. 5A). In addition, the phenotypes of EH were significantly correlated to LAE and PH, respectively, in the F2:3 population (Fig. 5B). The heritability (H2) estimates in the individual environment ranged from 79.195 to 92.14% for PE, from 79.43 to 91.21% for EH, from 76.85 to 867.8% for LAE and from 68 to 92.12% for ILE (Table 1). Overall, the maize plants clearly showed considerable natural variation in PH, EH, LAE and ILE and displayed an abundant genetic diversity.

**Table 1:** Descriptive statistics and broad-sense heritability for the leaf angle and internode length above the primary ear (LAE and ILE), plant height (PH), and ear height (EH)

Traits	Environment	Mean ± SD	Range	CV	Skew	kurtosis	H^2^
PH (F_2:3_)	2016 Changchun	227.67 ± 2.43	135–300	1.067%	-0.337	-0.500	87.95%
	2017 Changchun	225.81 ± 2.18	143–297	0.963%	-0.345	-0.683	85.45%
	2017 Gongzhuling	232.35 ± 2.24	155–345	0.964%	0.239	0.110	86.08%
	2018 Changchun	225.32 ± 1.76	157–300	0.781%	-0.025	-0.132	79.195%
	2018 Gongzhuling	229.96 ± 1.99	157–292	0.865%	-0.353	-0.549	82.93%
PH (RIL)	2019 Changchun	213.68 ± 1.55	144–286.8	0.73%	-0.09	0.228	89.1%
	2019 Gongzhuling	196.78 ± 1.65	132.4–270	0.84%	-0.163	0.048	90.1%
	2020 Changchun	195.33 ± 1.38	130–250	0.71%	-0.124	0.328	86.57%
	2020 Gongzhuling	207.83 ± 1.9	137–313	0.91%	0.589	1.035	92.14%
EH (F_2:3_)	2016 Changchun	100.26 ± 1.80	43–179	1.795%	0.350	-0.305	91.21%
	2017 Changchun	96.00 ± 1.10	55–145	1.145%	0.351	0.285	79.43%
	2017 Gongzhuling	92.59 ± 1.31	53–136	1.414%	0.324	-0.661	84.65%
	2018 Changchun	93.70 ± 1.13	55–136	1.205%	0.086	-0.491	80.36%
	2018 Gongzhuling	96.91 ± 1.21	59–165	1.248%	0.417	0.423	82.13%
EH (RIL)	2019 Changchun	76.49 ± 0.94	35–110.4	1.23%	-0.153	0.083	86.35%
	2019 Gongzhuling	83.19 ± 0.99	47–137	1.20%	0.234	0.575	87.66%
	2020 Changchun	82.2 ± 1.05	35–127	1.28%	-0.312	0.593	88.86%
	2020 Gongzhuling	91.11 ± 1.11	55–143	1.22%	0.212	0.179	89.7%
LA (F_2:3_)	2016 Changchun	28.71 ± 0.53	14–54	1.85%	0.539	0.116	82.64%
	2017 Changchun	31.53 ± 0.49	15–54	1.55%	0.562	0.254	76.85%
	2017 Gongzhuling	30.39 ± 0.59	13–55	1.94%	0.494	-0.395	82.35%
	2018 Changchun	29.98 ± 0.63	16–56	2.1%	0.244	0.415	83.16%
	2018 Gongzhuling	30.06 ± 0.58	14–55	1.93%	0.579	-0.185	81.15%
LA (RIL)	2019 Changchun	13.54 ± 0.11	9.93–18.5	0.81%	0.041	-0.053	86.5%
	2019 Gongzhuling	12.3 ± 0.08	8.22–15	0.69%	-0.291	0.161	82%
	2020 Changchun	11.6 ± 0.08	8.75–16.5	0.65%	0.373	1.195	86.8%
	2020 Gongzhuling	12.63 ± 0.11	9.12–19.5	0.87%	0.768	1.404	84.4%
IL (F_2:3_)	2016 Changchun	11.67 ± 0.13	7.7–20	1.11%	1.394	3.676	81.6%
	2017 Changchun	11.49 ± 0.13	3–20	1.13%	0.94	5.709	81.67%
	2017 Gongzhuling	12.11 ± 0.12	7.2–20	0.99%	1.264	3.764	79.38%
	2018 Changchun	14.40 ± 0.21	9–22	1.46%	0.391	-0.345	92.12%
	2018 Gongzhuling	14.09 ± 0.18	8–22	1.28%	0.872	1.226	89.87%
IL (RIL)	2019 Changchun	12.3 ± 0.08	15.2–39	1.41%	0.203	-0.376	81.6%
	2019 Gongzhuling	13.54 ± 0.11	10.67–50.83	1.7%	0.83	1.836	71.3%
	2020 Changchun	12.63 ± 0.11	17–47	1.27%	0.126	0.059	68%
	2020 Gongzhuling	11.6 ± 0.08	8.7–43.7	1.55%	0.111	0.304	90.7%

### *QTL* analysis

**Fig. 6:**
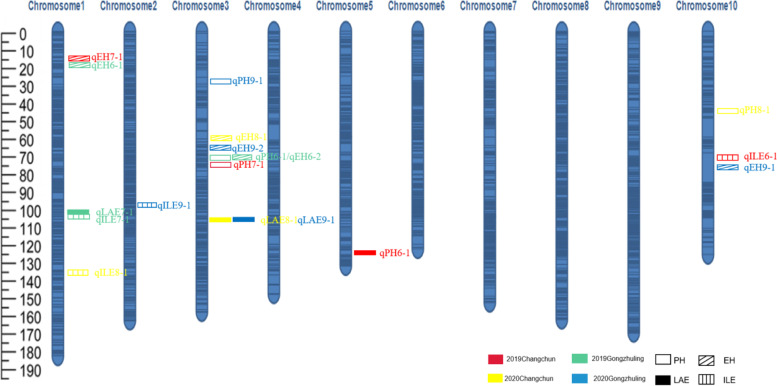
Plant architecture-related *QTL* detected for the RIL population in the four environments

Through *QTL* mapping, 60 *QTL* were co-located in 9 environments in the two populations. The LOD values ranged from 2.181 to 26.608, and the phenotypic contribution rates ranged from 0.734 to 30.748% (Table S2, Table 2, Figure S4, Fig. 6).

**Table 2:** Main effect *QTL* (R^2^ > 10%) for plant architecture traits in the F2:3 population

Trait	Environment	QTL	Chr	Marker interval	Position(cM)	Position(bp)	LOD	Add	R^2^(%)
PH	2016 Changchun	qPH1-1	3	umc2269-bnlg1505	331.5- 336.5	156,958,516–162,111,302	11.28	23.27	21.96
	2017 Changchun	qPH2-1	3	umc2265-umc1839	296.5–313.5	157,012,964–162,111,302	11.89	21.56	20.72
	2018 Gongzhuling	qPH5-1	9	umc109-umc1170	309.5–336.5	2,940,677–12,687,973	4.1145	13.51	11.5094
EH	2017 Changchun	qEH2-1	1	umc1723a-bnlg1803	23.5–39.5	12,448,970–28,638,545	7.18	9.33	11.43
	2017 Gongzhuling	qEH3-1	3	umc2265-umc1839	306.5–319.5	157,012,964–162,111,302	7.19	9.08	13.38
	2018 Changchun	qEH4-1	10	umc1824c-umc1589	411.5- 426.5	95,315,410–110,657,182	8.24	-11.57	16.21
	2018 Gongzhuling	qEH5-2	3	umc2265-umc1839	296.5–314.5	157,012,964–162,111,302	7.30	8.59	10.23
		qEH5-3	4	umc2287-umc1371	32.5- 42.5	138,472,340–236,999,463	6.49	6.18	10.53
LA	2017 Changchun	qLA2-2	3	umc2268- umc1641	376.5–385.5	184,713,010–230,956,846	13.898	5.905	24.226
	2018 Changchun	qLA4-1	3	umc2268-umc1641	376.5–385.5	184,713,010–230,956,846	5.097	4.583	10.413
IL	2018 Changchun	qIL4-2	10	phi054-umc2705	130.5–135.5	31,183,136–59,002,720	26.608	-2.812	30.748
		qIL4-3	10	umc1824c-umc1589	418.5–432.5	95,315,410–110,657,182	13.989	-2.260	19.545
	2018 Gongzhuling	qIL5-2	10	umc1824c-umc1589	420.5–428.5	95,315,410–110,657,182	12.401	-3.495	23.418

For PH, 7 *QTL* were identified for the F_2:3_ family population, 4 *QTL* were identified for the RIL population, and the phenotypic contribution rates ranged from 1.9% to 21.96%. On chromosome 3, the qPH1-1 and qPH2-1 located in the F2:3 family overlapped with the qPH6-1 and qPH7-1 located in the RIL population at the physical positions from 160,148,616–161,096,774 bp and 157,895,072–157,924,932 bp. Therefore, these two physical regions are considered to coincide with two *QTL* that exist stably in the three environments, and the phenotypic contribution rates ranged from 6.809–21.96%. A *QTL* that existed stably in three environments was also found on chromosome 10 at the physical position from 37,215,409–41,540,766 bp, with phenotypic contribution rates ranging from 2.08–14.635%. The additive effect value indicated that S122 increased alleles in the two physical regions from 160,148,616–161,096,774 bp and 157,895,072–157,924,932 bp, while Q102 and H132 increased alleles in the 37,215,409–41,540,766 bp physical region.

For EH, a total of 13 *QTL* were detected on chromosomes 1, 2, 3, 4, and 10, with the phenotypic contribution rates ranging from 3.92 to 13.38%. qEH3-1, qEH5-2 and qEH6-2 located on chromosome 3 were located in the same physical region from 160,148,616–161,096,774 bp and overlapped with the consistent *QTL* position for PH. Therefore, this position represents a pleiotropic *QTL* locus. Two major *QTL* (PVE > 10), qEH2-1 and qEH6-1 on chromosome 1, were located in the same physical region from 12,642,577–14,705,530 bp with a span of 2.1 Mb, with PVEs of 11.43 and 10.085%.

For LAE, 19 *QTL* were identified, accounting for 0.6–24.23% PVE. Two major *QTL* (qLA4-1 and qLA2-2) were mapped on chromosome 3. qLA4-1 (10.41%) and qLA2-2 (24.23%) were detected in the same place (Changchun) but in different years. The physical region of the two major *QTL* overlapped with that of another 3 *QTL* (qLA1-1, qLA8-1, and qLA9-1) from 186,194,870–187,045,576 bp with a span of 850,706 bp. The phenotypic contribution rates of qLA1-1, qLA8-1 and qLA9-1 were 0.759%, 8.677%, and 6.882%, respectively. The additive effect value indicated that Q102 and H132 increased alleles in the 186,194,870–187,045,576 bp physical region.

For ILE, 13 *QTL* were identified, accounting for 0.793–30.748% of PVE. One consistent *QTL* (qIL4-3, qIL5-2, and qIL6-1) region was detected in more than two environments and located in the physical position from 95,737,789–107,262,109 bp on chromosome 10, spanning 11 Mb, which was detected in 2018 in Changchun, 2018 in Gongzhuling and 2019 in Changchun with PVEs of 19.545%, 23.418% and 9.861%, respectively. Donor alleles for increased trait measurements came from ‘Q102’ and ‘H132’. Another consistent *QTL* region (qIL4-2 and qIL5-1) was also detected on chromosome 10, accounting for 30.748% and 6.710% of PVE, respectively. However, this consistent *QTL* region was only detected in the F2:3 population. Additionally, qIL2-1 overlapped with the region detected for qIL7-1 on chromosome 1.

### Functional annotation in three stable *QTL* regions

Functional annotation of genes in stable *QTL* regions in multiple environments helps to reveal the biological functions of genes. The stable *QTL* related to the LAE were located in the 186,194,870–187,045,576 bp region of chromosome 3, and 54 genes were annotated (Table S3). A stable pleiotropic *QTL* that simultaneously correlates PH and EH was located on chromosome 3 from 158,614,260–161,096,774 bp, with 442 genes annotated (Table S3). The stable *QTL* located in the 95,737,789–107,262,109 bp region of chromosome 10 was related to the ILE, with 1376 genes annotated (Table S3).

**Fig. 7:**
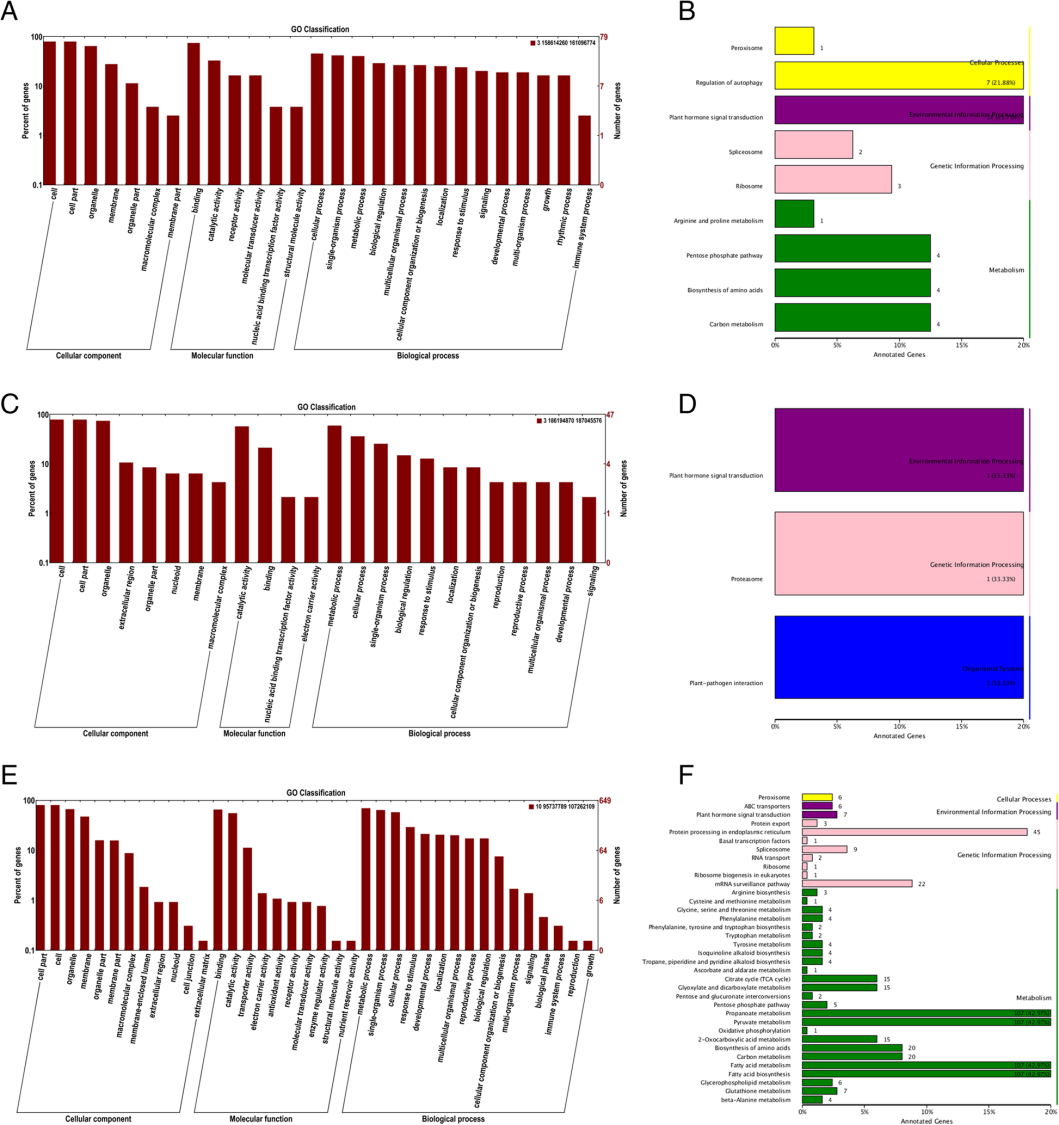
GO and KEGG enrichment of major *QTL* region genes. **A** GO enrichment in the 157,895,072–157,924,932 bp region of chromosome 3. **B** KEGG enrichment [31–33] in the 157,895,072–157,924,932 bp region of chromosome 3. **C** GO enrichment in the 186,194,870–186,203,649 bp region of chromosome 3. **D** KEGG enrichment [31–33] in the 186,194,870–186,203,649 bp region of chromosome 3. **E** GO enrichment in the 95,737,789–107,262,109 bp region of chromosome 10. **F** KEGG enrichment [31–33] in the 95,737,789–107,262,109 bp region of chromosome 10

Previous studies have provided useful information for understanding the possible mechanism of maize plant architecture traits. The role of hormones in plant morphogenesis has been widely confirmed, among which IAA, GA and BR play important roles. There is a gene, Zm00001d043000 (GO:0,008,152), on chromosome 3 from 186,194,870–187,045,576 bp that encodes the gibberellin receptor protein GID1, which is involved in activating the gibberellin signaling pathway (Fig. 7A and B, Table S3). There is also a gene, Zm00001d042292 (GO:0,009,734), on chromosome 3 from 158,614,260–161,096,724 bp that encodes the auxin response protein SAUR50, which is involved in activating the auxin signaling pathway (Fig. 7C and D, Table S3). Additionally, there is a gene, Zm00001d025008 (GO:0,009,742), on chromosome 10 from 95,737,789–107,262,109 bp that is involved in the brassinolide-mediated signaling pathway (Fig. 7E and F, Table S3). These 3 candidate genes are worthy of further study to determine their role in maize morphogenesis.

### Candidate gene expression analysis

**Fig. 8:**
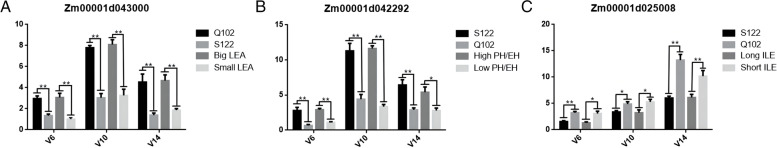
Relative expression levels of three candidate genes in phenotypically different plant samples from the maize at three growing stages analyzed via qRT-PCR. **A** The expression levels of the LAE-related candidate gene Zm00001d043000 at V6, V10 and V14 stages, respectively. **B** The expression levels of the PH/EH-related candidate gene Zm00001d042292 at V6, V10 and V14 stages, respectively. **C** The expression levels of the ILE-related candidate gene Zm00001d025008 at V6, V10 and V14 stages, respectively. **, and * specify the significance at the levels of *P* < 0.01, and *P* < 0.05, respectively

In order to explore the function of candidate genes, the parents of RIL and progeny individuals (Table S6) with significant phenotype differences were used to analyze the expression levels of candidate genes. The analysis results showed that the expression levels of the candidate genes were significantly different between the parents and also showed similar differences in the individual offspring. The expression level of Zm00001d043000 in Q102 and plants with large leaf angles was significantly higher than that in S122 and plants with small leaf angles, and the expression level was the highest in the V10 stage (Fig. 8A). The expression pattern of Zm00001d042292 was similar to that of Zm00001d043000. In plants with obvious phenotypic differences, the expression level was also significantly different, and the expression level was higher in male parent S122 and its phenotypically similar progeny. (Fig. 8B). The expression pattern of Zm00001d025008 was completely different. Although the expression level of plants with significant differences in appearance also showed significant differences, the expression level of Zm00001d025008 was higher in plants with shorter internodes (Fig. 8C). In addition, the highest expression level of Zm00001d025008 appeared in the V14 period, and the expression level increased with the continuous growth of the plant. The significant differences between the parents of the candidate genes were consistent with the phenotypic differences and showed prominent expression changes during the critical growth period for phenotype formation (Table S6, Figs. 4 and 8). In individuals with recombinant inbred lines inheriting the parental phenotype, the candidate genes showed the same expression pattern as the parent.

## Discussion

This study describes two *QTL* mapping populations, with the first being a parent population consisting of 217 F2:3 families, and a genetic map with 171 SSR molecular markers covering 10 chromosomes was drawn (Fig. 2). Another parent population composed of 209 recombinant inbred lines was identified with a high density genetic map based on SNP molecular markers (Fig. 3A). We used these two genetic maps to identify the PH, EH, LAE and ILE of maize in Changchun and Gongzhuling for 5 years, and a total of 60 *QTL* were mapped (Table S2). Among them, there are 11 *QTL* related to PH, 13 *QTL* related to EH, 23 *QTL* related to the LAE, and 13 *QTL* related to the ILE. Among the 11 *QIL* related to PH, we found two *QTL* that were consistent in multiple environments in the 160,148,616–161,096,774 bp and 157,895,072–157,924,932 bp regions, respectively, of chromosome 3. The 157,895,072–157,924,932 bp region corresponded to a *QTL* co-located for PH and EH in Bin3.05, which was a hotspot area for *QTL* related to PH and EH [34–36]. In the correlation analysis of phenotypic traits, we found that there was a significant correlation between PH and EH (Fig. 5). Many previous studies also confirmed that PH and EH are related [37]. Interestingly, we also found that there was a significant correlation between the LAE and PH (Fig. 5A). Among the *QTL* related to LAE, we found a stable and consistent *QTL* from 186,194,870–186,203,649 bp that exists in 5 environments at the same time, which can explain 24% of the phenotypic variation, located in Bin3.06. In Bin3.06, there are many *QTL* and meta-*QTL* related to PH, leaf angle and yield, and the broad heritability of LAE and PH is high, indicating that the genetic locus controlling the two traits is less affected by the environment and it may be interlocked [38]. In contrast, there is a correlation between IL and PH in many studies, and both IL and PH are mainly affected by additive effects. However, this study did not find a significant correlation between ILE and PH. The generalized heritability of ILE ranges from 68 to 92.12% in 9 environments (Table S2), and the phenotype is easily affected by the environment. Therefore, different environments and genetic backgrounds may be the reason for this discrepancy. In addition, we discovered a new consistent *QTL* region (95,737,789–107,262,109 bp) related to ILE, which was co-located in three environments at the same time, and the phenotypic contribution rate reached up to greater than 30%. This is a major *QTL*, in which there may be key genes that affect ILE (Table S2).

Although there have been an increasing number of studies on maize plant architecture traits in recent years, they are not in-depth. A large number of studies have shown that PH, EH, LA and IL are mainly regulated by hormones, involving GA and BR synthesis, transport and signal transduction pathways. At the same time, IAA, the main factor that determines internode elongation by activating cell elongation, has also been found to be involved in PH regulation [39–41]. In this study, we conducted gene mining on the three consistent *QTL* (157,895,072–157,924,932 bp, 186,194,870–186,203,649 bp, and 95,737,789–107,262,109 bp), and performed functional annotations on the genes of the three *QTL* regions. A responsive auxin protein SAUT50 gene (Zm00001d042292) involved in the auxin activation signaling pathway was selected from the consistent *QTL* (157,895,072–157,924,932 bp) related to PH and EH. The SAUR gene family was originally defined as a set of auxin-inducing genes that regulate development, especially in hypocotyls, many of which promote plant growth by elongating cells. In the analysis of the expression level of SAUT50, we found that the expression level of SAUT50 in individuals with high PH was significantly higher than that in individuals with low PH, and the expression level of SAUT50 peaked during the jointing stage (V10) of maize, indicating that the formation process of SAUT50 played an important role in determining maize plant height (Fig. 8B). Similar to IAA, BR also changes the PH by extending the internode length. BR is the main endogenous hormone that promotes internode elongation and is used to regulate plant dwarfing. At present, 13 genes related to BR synthesis, including DET2, Dwarf1, Dwarf4, etc., and 4 genes related to BR signaling, BAK1, BRS1, Bri and Bri2, have been cloned in the model plant Arabidopsis [42, 43]. Genes involved in BR synthesis and abnormal signal transduction have also been found in rice, cucumber, corn, rapeseed and other crops. Analysis of an annotated gene, Zm00001d025008, involved in the brassinolide-mediated signaling pathway in the consistent *QTL* related to ILE showed that its expression level in individuals with long ILE was significantly lower than that in individuals with short ILE, and the expression level continued to increase with plant growth until the plant transformed from vegetative growth to reproductive growth (Fig. 8C). Zm00001d025008 may play a negative regulatory role in the brassinosteroid-mediated signaling pathway, which is consistent with the negative additive effect of the *QTL* where the gene is located. We screened a gene GID1 (Zm00001d043000) related to GA regulation in the consistent *QTL* region related to the LAE, and the expression of the GID1 gene in individuals with a large LAE was significantly higher than that in individuals with a small LAE (Fig. 8A). The maize leaf angle is mainly determined by the size of leaf occipital cells [44–46]. Cytological observations of upright leaves in rice showed that the growth of cells at the occipital of the upright leaves was inhibited, and the cell length became shorter, resulting in a smaller leaf angle [47]. In contrast, the cells at the proximal end of the occiput of the flat leaf blade were slender, causing the leaf to bend and sag with a larger leaf angle. In 2007, Ueguchi-Tanaka et al. first identified the GA receptor protein Gibberellin insensitive dwarf1 (GID1) from rice and confirmed that GID1 is a soluble receptor that mediates GA signaling in rice [48]. In the GA signal transduction pathway, the DELLA protein mainly acts as a repressor and inhibits plant growth and development, while GA promotes plant growth and development by degrading the DELLA protein. GA uses the GA-GID1-DELLA complex mode of action [49]. The receptor protein GID1 senses and binds to GAs, interacts with the N-terminus of the DELLA protein, and degrades the DELLA protein through the ubiquitination/26S protease pathway; thus, the leaf occipital cells become longer and the leaf angle increases [50].

## Methods

### Plant materials

The F2:3 family bi-parent population, which was developed from a cross between the flat maize inbred line H132 and the compact line S122, was used to map *QTL* (Fig. 1A). The female parent H132 is a self-selected line. H132 is an improved inbred line of Dan988 from the Plant Technology Center of Jilin Agricultural University. S122 comes from a self-selected line of the NIL population constructed by H201 × Dan 340 and the reincarnation parent Dan 340, provided by the Liaoning Liangyu Seed Industry Co., Ltd. The F2:3 population contained 217 F2:3 families developed by self-pollinating 217 F2 individuals. In 2016, the 217 F2 individuals, the two parent lines, and the F1 generation were planted in Changchun (N: 43°05′/E: 124°18′), Jilin Province. In 2017 and 2018, the F2:3 families were planted in Gongzhuling (N: 43°31′/E: 124°49′) and Changchun, Jilin Province. At each location, the field experiment had a randomized complete block design with three replications.

In the high-density genetic map *QTL* mapping experiment, the recombinant inbred line was obtained by crossing the flat inbred line Q102 and the inbred line S122 (Fig. 1A). Q102 is a self-selected line from a recombinant inbred line constructed from a local variety in Northeast China provided by the Qiufeng Agricultural Research Institute, Huadian, Jilin Province. The recombinant inbred line population contained 209 inbred lines, which were formed by 209 F2 individuals who were self-bred continuously for 6 generations by the single seed method. In 2019 and 2020, the RIL populations were planted in Gongzhuling and Changchun, Jilin Province. At each site, the field experimental design was consistent with the F2:3 population. The phenotypic data of the F2:3 and RIL populations are shown in Table S1. Study protocol comply with relevant institutional, national, and international guidelines and legislation.

### Phenotypic data collection and analysis

The following traits related to plant architecture were evaluated: PH, EH, LAE, and ILE. PH was measured from the ground to the tip of the primary inflorescence (Fig. 1B), while EH was measured from the ground to the node of the primary ear (Fig. 1B). LAE was measured between the stem and leaf above the primary ear (Fig. 1B), and ILE was measured from a node to the next node between the tip of the primary inflorescence and the primary ear (Fig. 1B). In each line, ten consecutive plants were selected for the PH, EH, LAE and ILE measurements. The phenotype of each line was obtained by averaging the phenotypic values of the ten measured plants. Descriptive statistical analyses, frequency distribution analyses, analysis of variance (ANOVA), and correlation analyses were performed using SPSS Statistics 25 software (http://www.ibm.com/legal/copytrade.shtml) (Figure S2, Table S2, Fig. 5). The broad-sense heritability was calculated according to our previous method [51].

$${\mathrm{H}}^{2}= {{\updelta }^{2}}_{\mathrm{G}}/{{\updelta }^{2}}_{\mathrm{P}}, {{\updelta }^{2}}_{\mathrm{G}}= (\mathrm{MSG }-\mathrm{ MSE}/\mathrm{rep})$$

$${{\updelta }^{2}}_{\mathrm{P}} = (\mathrm{MSG }-\mathrm{ MSE}/\mathrm{rep}) +\mathrm{ MSE}$$

In the formula, δ^2^_G_ is the variance of the genotype, δ^2^_P_ is the variance of the phenotype, MSE is the mean square error, MSG is the mean square genotype, and rep (rep = 3) is the number of repetitions of each experiment.

### DNA extraction and genotyping

The DNA was extracted from the plant materials as described by Mu et al. [52]. Simple sequence repeat (SSR) markers covering the entire genome were selected from the maize genome database (http://www.maizegdb.org/) and screened to identify those that were polymorphic between the two parents (H132 × S122). All SSR marker primers were synthesized by the Kumei Biotech Co., Ltd. These markers were used to genotype the mapping population (F2 population). Genotyping was performed by a QIAGEN capillary electrophoresis instrument.

The RIL population was genotyped using the SLAF-seq method. SLAF tags were designed based on the Zm-B73-REFERENCE-GRAMENE-4.0 reference genome (https://www.maizegdb.org/genome/assembly/Zm-B73-REFERENCE-GRAMENE-4.0). To obtain more than 246,254 SLAF tags per genome (defined as an enzyme fragment sequence of 414–464 bp), bioinformatics software was used to predict the restriction enzyme digestion of the reference genome and select the most suitable restriction enzyme digestion plan. The selection criteria were as follows: (1) the proportion of digested fragments located in the repetitive sequence was as low as possible; (2) the digested fragments were distributed as evenly as possible in the genome; (3) there was consistency between the length of the digested fragments and the specific experimental system; and (4) the number of fragments (SLAF tags) finally obtained met the expected number of tags. Two restriction enzymes HeaIII and Hpy166II were used to digest the DNA. A was added to the 3′ end of the obtained fragment (SLAF tag), the Dual-index sequencing adapter was connected, and PCR amplification, purification, mixing, and gel cutting to select the target fragment were performed. After the library quality inspection, the SLAFs were sequenced using the Illumina HiSeq 2500 paired-end sequencing platform. To evaluate the accuracy of the database construction experiment, Nipponbare rice (*Oryza sativa* L. *japonica*) was selected as a control (Control) and subjected to the same treatment for database construction and sequencing. Low-quality reads were filtered out, and BWA software was used to map the filtered reads to the reference genome. Sequences with a similarity greater than 95% were considered the same SLAFs. All of the SLAF markers that were consistent in parents and offspring were genotyped.

### Construction of the genetic map and *QTL* mapping

A genetic linkage map was constructed based on previous descriptions. Based on deep resequencing of parents, SLAFs were verified by allele source. A high-density genetic map was constructed with polymorphic SLAFs whose parental genotype was aa × bb and whose progeny genotype was ab or misses. All SLAF markers were grouped according to the chain LOD threshold of 3.0, and the position and order of the grouping markers were arranged using the est.map function in the R/qtl package. Combining the phenotypic data of the RIL population, the compound interval mapping in the R/qtl package was used to identify *QTL*. The LOD thresholds of significant *QTL* were determined with 1000 permutations and a P value of 0.05 using the mqmpermotation function in R/qtl.

For the F2 population (H132 × S122), a genetic map was constructed using the software *QTL* ICIMapping version 4.2 (http://www.isbreeding.net). A LOD threshold of 3.0 was used to assign markers to the same linkage group. The observed frequencies at each marker were tested against the expected Mendelian segregation ratio of 1:2:1 using a chi-squared test for goodness of fit. Then, the QTLs for the traits of plant architecture were identified using the inclusive composite interval mapping method implemented in the software QTL ICIMapping version 4.2.

### Identification of candidate genes

To identify the putative genes, the physical position of the identified *QTL* was mapped to the Zm-B73-REFERENCE-GRAMENE-4.0 genome of the maize inbred line B73. GO and KEGG annotation were performed on the genes in the *QTL* region. Candidate genes were mined from the sequences of the identified *QTL* related to the plant architecture traits.

### Quantitative reverse transcription PCR

To verify the function of *QTL* candidate genes, 5 lines with the maximum phenotypic values and 5 with the minimum phenotypic values for the PH, EH, LAE and ILE were selected in the RIL population (Table S6). The 10 individuals were used to analyze the expression levels of the candidate genes along with the parents (S122, Q102).

Newly grown young leaf tissues were picked at the 6-leaf (6 V), 10-leaf (10 V), and 14-leaf (14 V) stages of maize growth for expression analysis. From 6 V, the leaf pillows could be seen, the stems were elongated, and the plant height increased. By 10 V, the leaf development rate was 1 leaf every 2–3 days. Rapid growth marked the 14 V stage, and this stage occurred about two weeks before flowering. These three periods reflect the process of maize morphogenesis. The primers used for qRT-PCR were designed by IDT online software (https://sg.idtdna.com/pages) (Table S6) and were synthesized by the Coome Biotech Co., Ltd. Using *ActinII *as an internal reference gene, the relative expression levels of candidate genes were calculated by comparison using the 2^−∆∆ct^ method. The qRT-PCR and data analysis were performed using methods described by Chen et al. [53].

## Conclusion

In this study, we systematically analyzed plant architecture traits and identified *QTL* for PH, EH, ILE, and LAE of maize in 9 environments. Three consistent *QTL* with stable main effects in multiple environments were obtained, three candidate genes were mined, and the expression levels of the candidate genes were analyzed. The study of plant architecture traits is of great significance to the cultivation of new high-quality and high-yield maize germplasm. In addition, the *QTL* mapping of LAE and ILE has enriched the research on the maize canopy, which is helpful for in-depth research on genetic improvement of the canopy structure. The mining of candidate genes and the analysis of their expression levels aid research on related mechanisms. These results laid the foundation for the molecular mechanism-, genetic- and marker-assisted breeding for maize plant architecture, especially for canopy structure morphogenesis.

## Supplementary Information

**Additional file 1:** **Figure S1.** The results of monomer source evaluation.

**Additional file 2:** **Table S1.** Two population phenotypic data.

**Additional file 3:** **Figure S2.**The freq uency distribution of, the plant height (PH), ear height (EH) and leaf angle and internode length above the primary ear (LAE,ILE). (A) Frequency distribution in the F2:3 family population. (B) Frequency distribution in the recombinant inbred line population. CC, and GZ represent Changchun, and Gongzhuling, respectively.

**Additional file 4:** **Figure S4. ** Quantitative trait loci (*QTL*) for plant architecture traits mapped in the F2:3 population.

**Additional file 5:** **Figure S3. **Marker linkage relationship results on linkage group. Each row and each column are Markers arranged in the order of the map, and each small square represents the recombination rate between the two Markers. The change in color from yellow to red to purple represents the change in recombination rate from small to large. The closer the Marker's recombination rate is, the closer the color is to yellow, and the farther the Marker's recombination rate is, the closer it is to purple.

**Additional file 6:** **Table S2. **Quantitative trait loci (*QTL*) for plant height(PE), ear height(EH), leaf angle and inernode length above the primary ear detected in different environments.

**Additional file 7:** **Table S3.** Confidence interval GO annotation.

**Additional file 8:** **Table S4.** Confidence interval KEGG annotation.

**Additional file 9:** **Table S5. **Names and data of phenotypic extreme value materials of plant architecture traits.

**Additional file 10:** **Table S6.** List of primers used for the qRT-PCR assay of the key genes involved in plant architecture traits.

## Data Availability

All data analyzed during this study are included in the supplementary information files, and genotypic data have been deposited in the Sequence Read Archive (https://www.ncbi.nlm.nih.gov/sra) under the accession number PRJNA778629.

## Abbreviations

SLAF: Specific length amplified fragment sequencing
*QTL*: Quantitative trait loci
SNP: Single nucleotide polymorphism
RILs: Recombinant inbred lines
SSR: Simple Sequence Repeat
H2: Broad-sense heritability
GO: Gene Ontology
PH: Plant height
KEGG: Kyoto Encyclopedia of Genes and Genomes
EH: Ear height
LAE: Leaf angle above the primary ear
ILE: Internode length above the primary ear
qPCR: Real-time Quantitative Polymerase Chain Reaction
