# Supplementary material for: Maize plant architecture trait QTL mapping and candidate gene identification based on multiple environments and double populations
Source: BMC Plant Biol. 2022 Mar 11;22:110. doi: 10.1186/s12870-022-03470-7 (PMC8915473; doi:10.1186/s12870-022-03470-7)
Supplement: Supplementary file 1 — Additional file 1: Figure S1. The results of monomer source evaluation. [file 12870_2022_3470_MOESM1_ESM.docx]

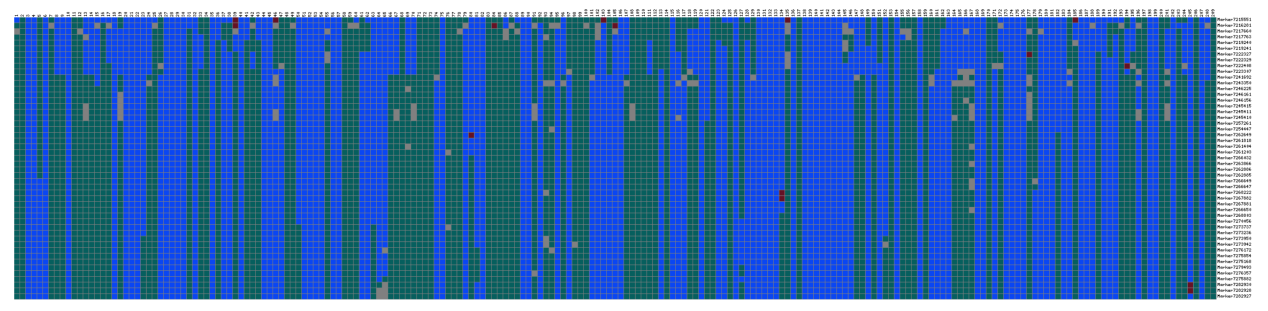


**Figure S1**: The results of monomer source evaluation. Each row represents a marker, arranged in order of position on the linkage group. Each column represents a chromosome in a sample, green represents those from the female parent, blue represents those from the male parent, and red represents the heterozygous type. The position where the color of the same column changes is the position where the recombination event occurred.
