# Supplementary material for: Maize plant architecture trait QTL mapping and candidate gene identification based on multiple environments and double populations
Source: BMC Plant Biol. 2022 Mar 11;22:110. doi: 10.1186/s12870-022-03470-7 (PMC8915473; doi:10.1186/s12870-022-03470-7)
Supplement: Supplementary file 3 — Additional file 3: Figure S2.The freq uency distribution of, the plant height (PH), ear height (EH) and leaf angle and internode length above the primary ear (LAE,ILE). (A) Frequency distribution in the F2:3 family population. (B) Frequency distribution in the recombinant inbred line population. CC, and GZ represent Changchun, and Gongzhuling, respectively. [file 12870_2022_3470_MOESM3_ESM.docx]

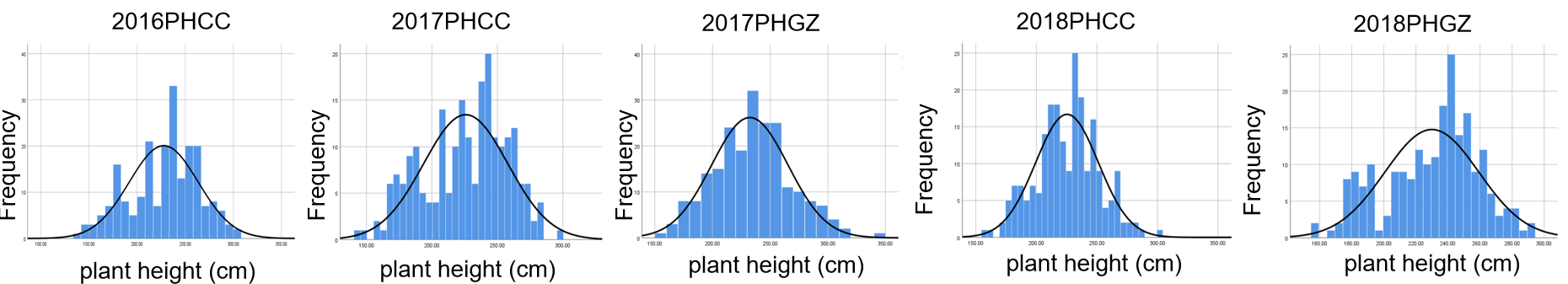


**A**


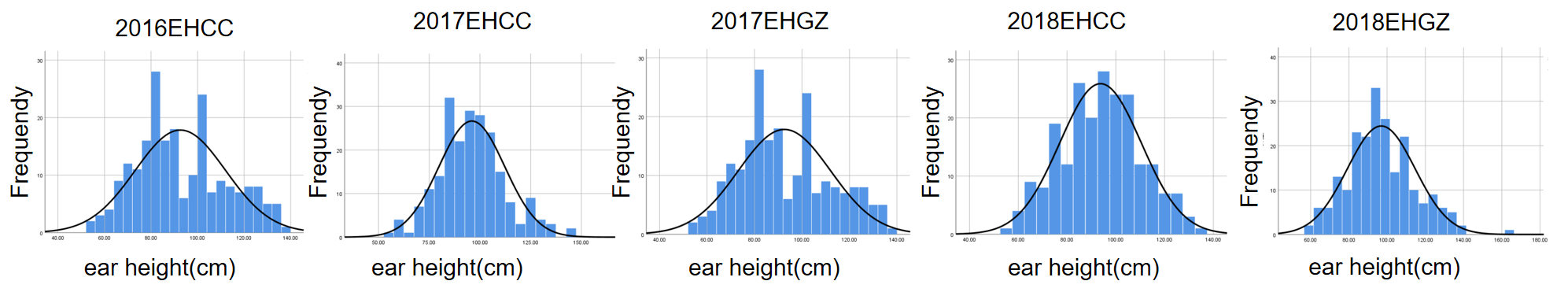


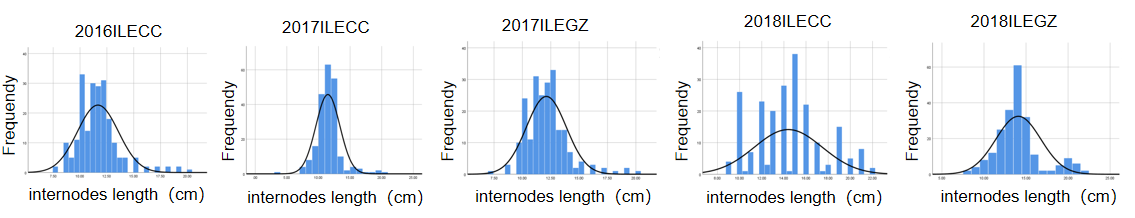


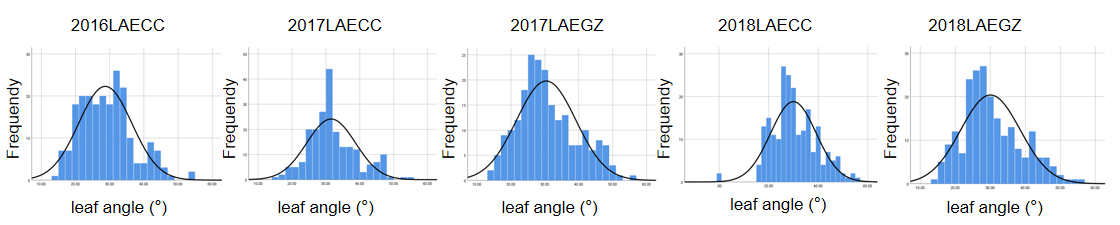


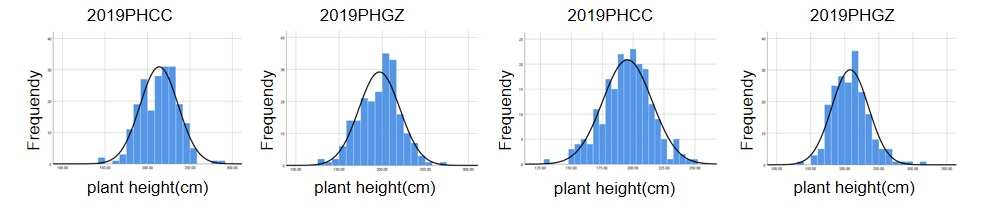


**B**


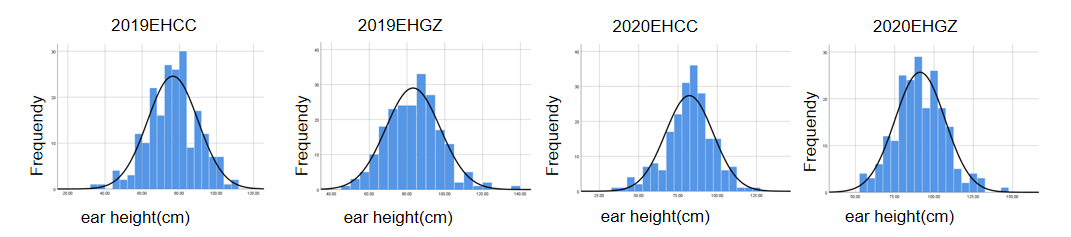


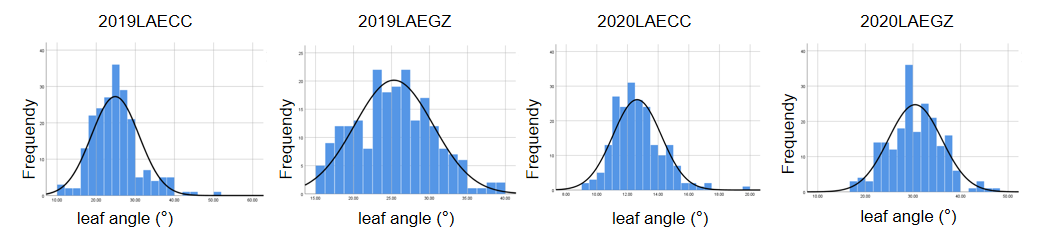


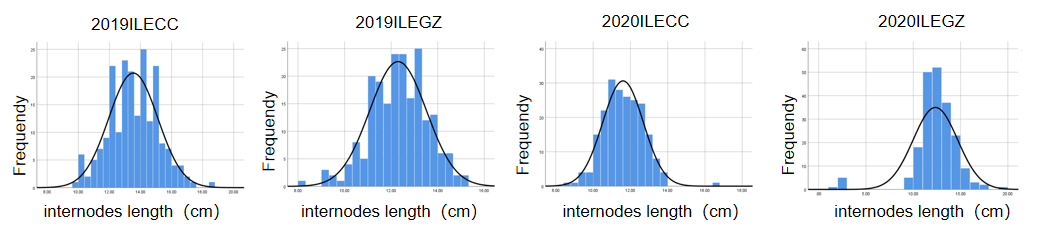
 **Figure S2:** The frequency distribution of, the plant height (PH), ear height (EH) and leaf angle and internode length above the primary ear (LAE,ILE). (A) Frequency distribution in the F2:3 family population. (B) Frequency distribution in the [recombinant](C:/Program%20Files%20(x86)/Youdao/Dict/8.9.9.0/resultui/html/index.html#/javascript:;) [inbred](C:/Program%20Files%20(x86)/Youdao/Dict/8.9.9.0/resultui/html/index.html#/javascript:;) [line](C:/Program%20Files%20(x86)/Youdao/Dict/8.9.9.0/resultui/html/index.html#/javascript:;) population. CC, and GZ represent Changchun, and Gongzhuling, respectively.
