# Supplementary material for: Maize plant architecture trait QTL mapping and candidate gene identification based on multiple environments and double populations
Source: BMC Plant Biol. 2022 Mar 11;22:110. doi: 10.1186/s12870-022-03470-7 (PMC8915473; doi:10.1186/s12870-022-03470-7)
Supplement: Supplementary file 4 — Additional file 4: Figure S4. Quantitative trait loci (QTL) for plant architecture traits mapped in the F2:3 population. [file 12870_2022_3470_MOESM4_ESM.doc]

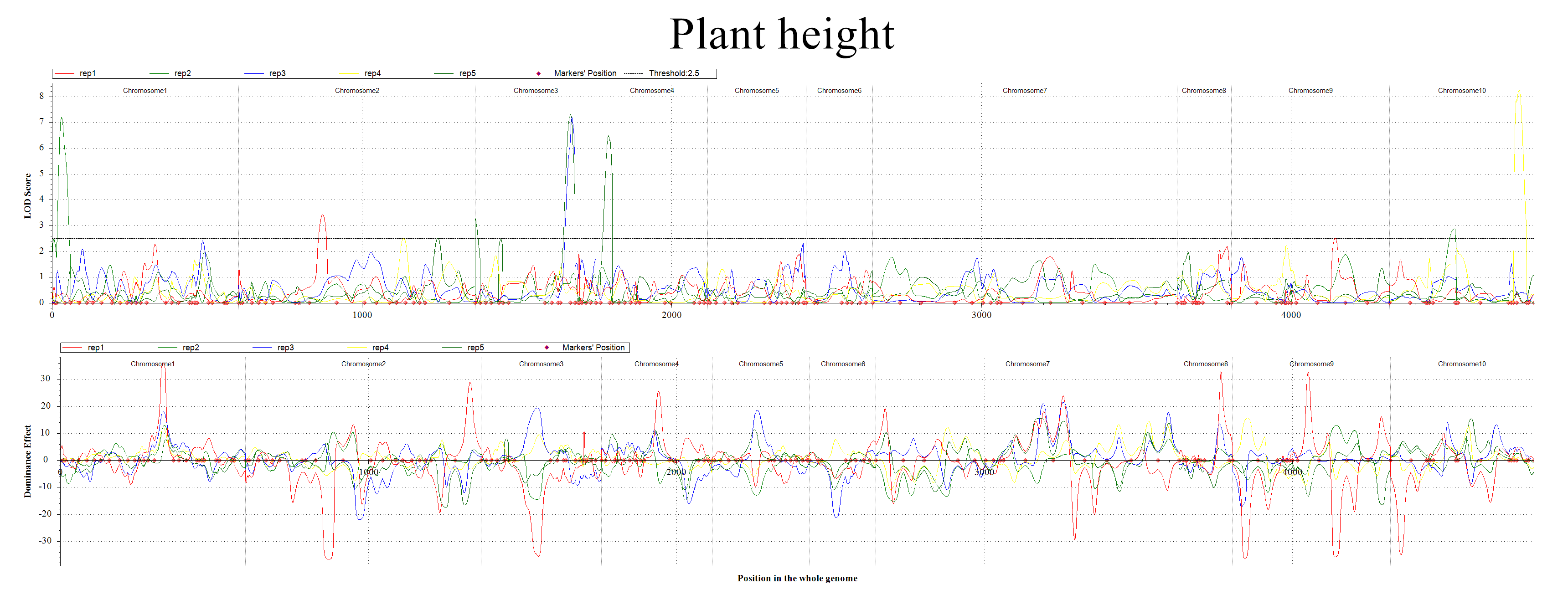


B

A


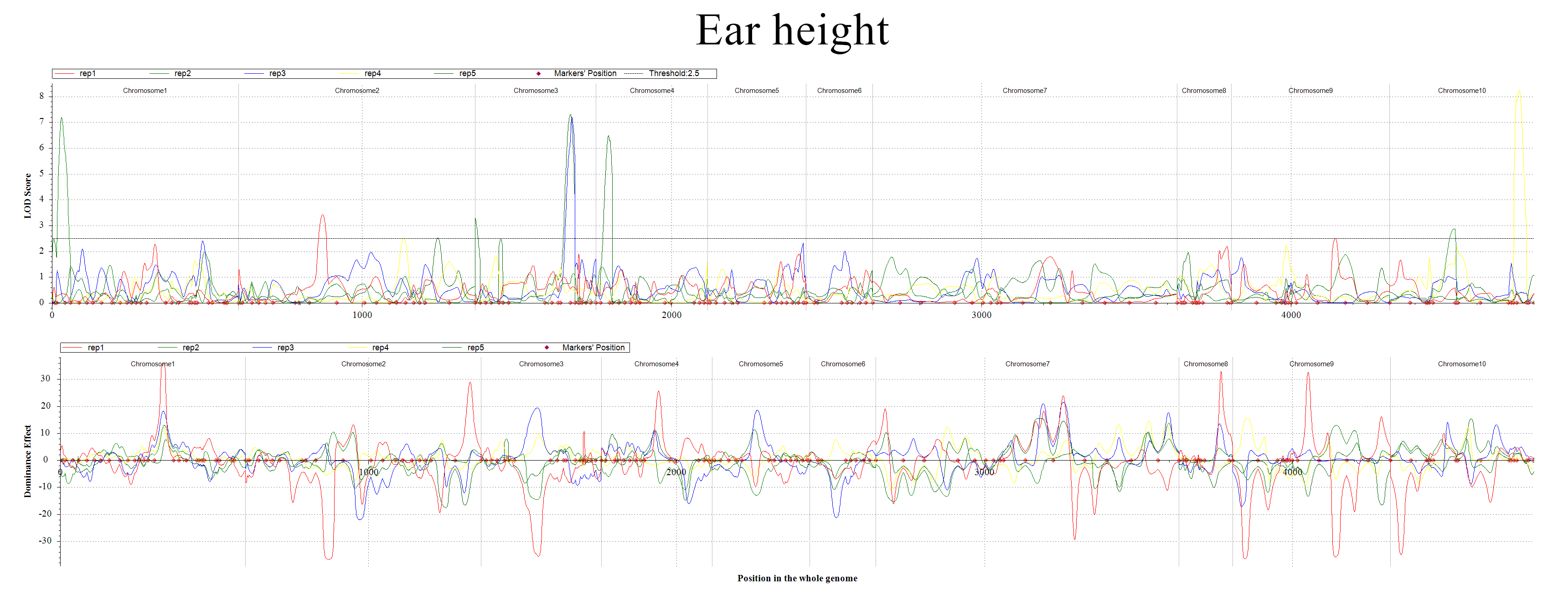


C


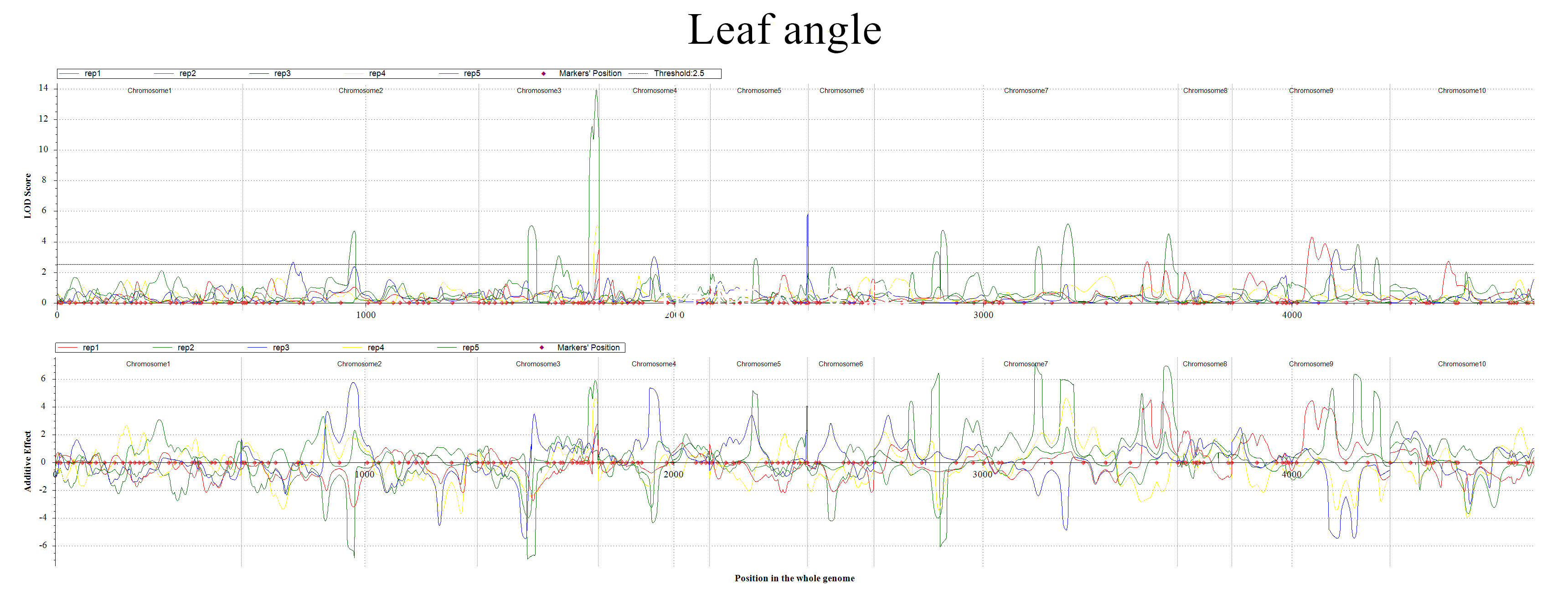


D


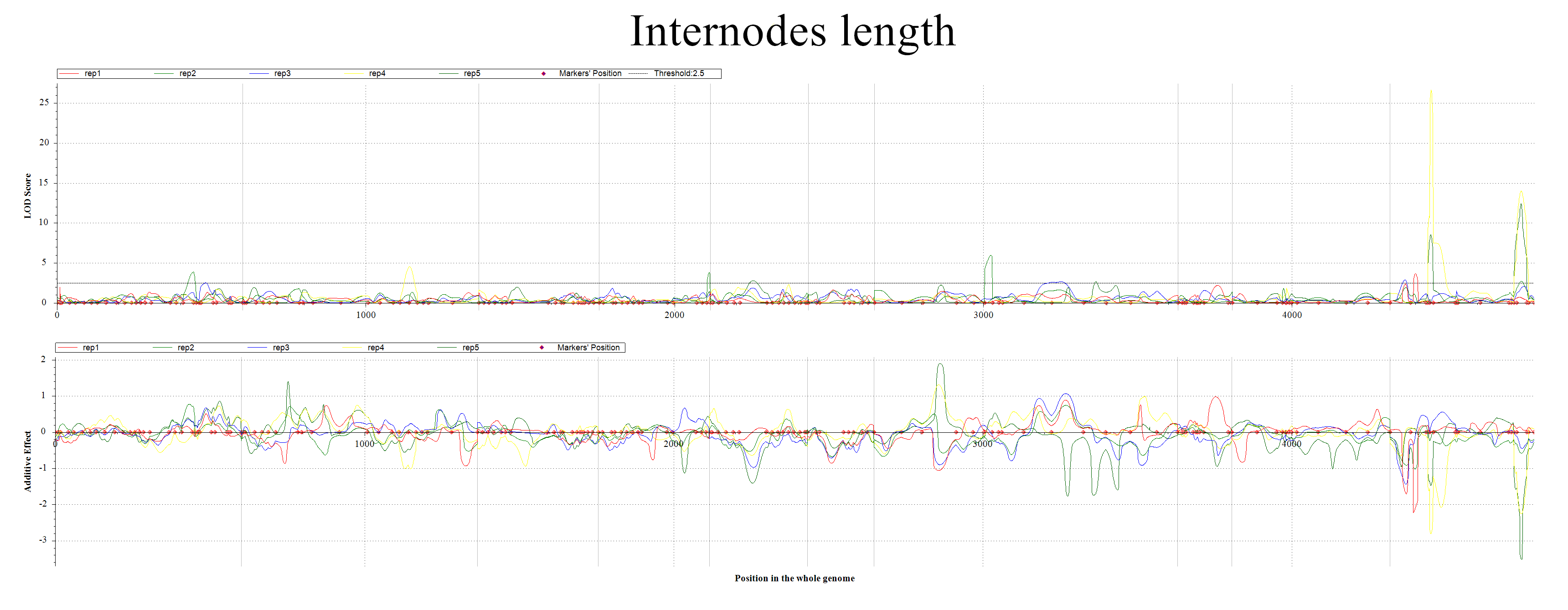


**Figure S4**: Quantitative trait loci (*QTL*) for plant architecture traits mapped in the F2:3 population. The x-axis shows the genetic position along the chromosomes. A vertical bar separates adjacent chromosomes. The y-axis represents the logarithm of the odds (LOD) score of each scanning position. The dotted line represents the LOD significance threshold. Green, red, blue, yellow and dark green represent, 2016 - Changchun, 2017 - Changchun, 2017 - Gongzhuling, 2018 - Changchun, and 2018 - Gongzhuling, respectively. A, B, C, and D represent the four plant architecture traits of plant height, ear height, leaf angle and internode length above the primary ear respectively.
