# Supplementary material for: Maize plant architecture trait QTL mapping and candidate gene identification based on multiple environments and double populations
Source: BMC Plant Biol. 2022 Mar 11;22:110. doi: 10.1186/s12870-022-03470-7 (PMC8915473; doi:10.1186/s12870-022-03470-7)
Supplement: Supplementary file 5 — Additional file 5: Figure S3. Marker linkage relationship results on linkage group. Each row and each column are Markers arranged in the order of the map, and each small square represents the recombination rate between the two Markers. The change in color from yellow to red to purple represents the change in recombination rate from small to large. The closer the Marker's recombination rate is, the closer the color is to yellow, and the farther the Marker's recombination rate is, the closer it is to purple. [file 12870_2022_3470_MOESM5_ESM.docx]

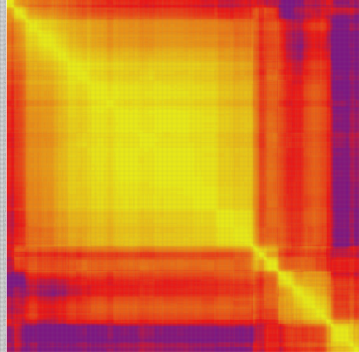

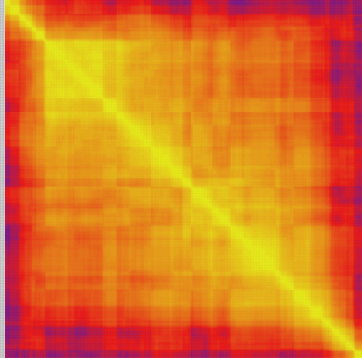

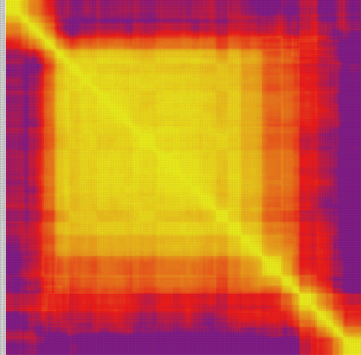


**[chromosome](C:/Program%20Files%20(x86)/Youdao/Dict/8.9.9.0/resultui/html/index.html#/javascript:;)1**

**[chromosome](C:/Program%20Files%20(x86)/Youdao/Dict/8.9.9.0/resultui/html/index.html#/javascript:;)2**

**[chromosome](C:/Program%20Files%20(x86)/Youdao/Dict/8.9.9.0/resultui/html/index.html#/javascript:;)3**

**[chromosome](C:/Program%20Files%20(x86)/Youdao/Dict/8.9.9.0/resultui/html/index.html#/javascript:;)6**

**[chromosome](C:/Program%20Files%20(x86)/Youdao/Dict/8.9.9.0/resultui/html/index.html#/javascript:;)5**

**[chromosome](C:/Program%20Files%20(x86)/Youdao/Dict/8.9.9.0/resultui/html/index.html#/javascript:;)4**


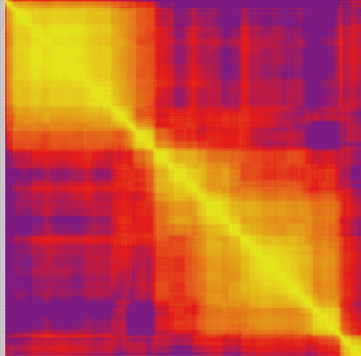

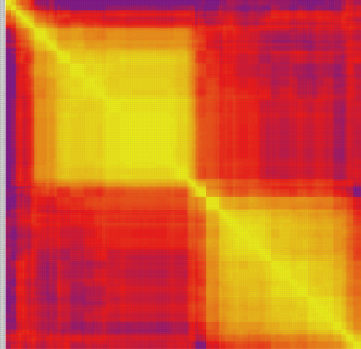

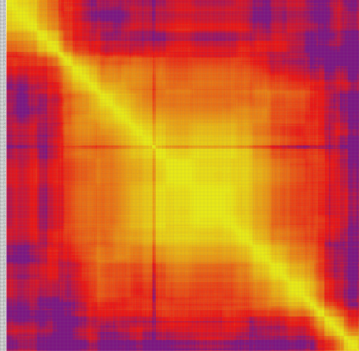


**[chromosome](C:/Program%20Files%20(x86)/Youdao/Dict/8.9.9.0/resultui/html/index.html#/javascript:;)7**

**[chromosome](C:/Program%20Files%20(x86)/Youdao/Dict/8.9.9.0/resultui/html/index.html#/javascript:;)8**

**[chromosome](C:/Program%20Files%20(x86)/Youdao/Dict/8.9.9.0/resultui/html/index.html#/javascript:;)9**


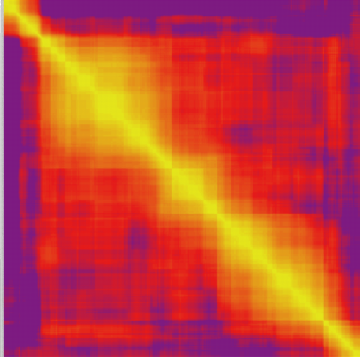

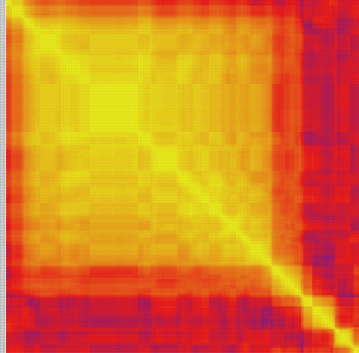

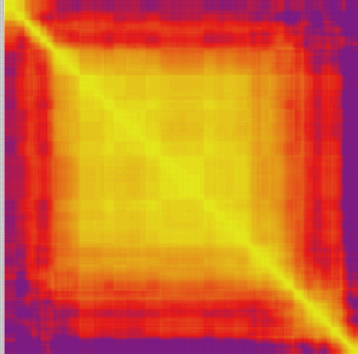


**[chromosome](C:/Program%20Files%20(x86)/Youdao/Dict/8.9.9.0/resultui/html/index.html#/javascript:;)10**


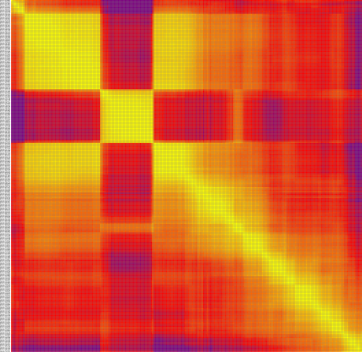


**Figure S3** : Marker linkage relationship results on linkage group. Each row and each column are Markers arranged in the order of the map, and each small square represents the recombination rate between the two Markers. The change in color from yellow to red to purple represents the change in recombination rate from small to large. The closer the Marker's recombination rate is, the closer the color is to yellow, and the farther the Marker's recombination rate is, the closer it is to purple.
