# Supplementary material for: Maize plant architecture trait QTL mapping and candidate gene identification based on multiple environments and double populations
Source: BMC Plant Biol. 2022 Mar 11;22:110. doi: 10.1186/s12870-022-03470-7 (PMC8915473; doi:10.1186/s12870-022-03470-7)
Supplement: Supplementary file 6 — Additional file 6: Table S2. Quantitative trait loci (QTL) for plant height(PE), ear height(EH), leaf angle and inernode length above the primary ear detected in different environments. [file 12870_2022_3470_MOESM6_ESM.docx]

**Table S2:** Quantitative trait loci (QTL) for plant height(PE)、ear height(EH)、leaf angle and inernode length above the primary ear detected in different environments.

| **Trait** | **Environment** | **QTL** | **Chr** | **Marker interval** | **Position(cM)** | **Position(bp)** | **LOD** | **Add** | **R^2^** |
| --- | --- | --- | --- | --- | --- | --- | --- | --- | --- |
| PH(F_2:3_) | 2016 Changchun | qPH1-1 | 3 | umc2269-bnlg1505 | 331.5- 336.5 | 156958516-162111302 | 11.28 | 23.27 | 21.96 |
|  | 2017Changchun | qPH2-1 | 3 | umc2265-umc1839 | 296.5-313.5 | 157012964- 162111302 | 11.89 | 21.56 | 20.72 |
|  | 2017Gongzhuling | qPH3-1 | 2 | umc1525- umc1934 | 349.5-373.5 | 10606800-233654880 | 3.19 | 10.70 | 9.96 |
|  |  | qPH3-2 | 10 | phi054-umc2705 | 132.5-161.5 | 31183136-59002720 | 6.0519 | -15.6131 | 2.1923 |
|  | 2018Gongzhuling | qPH5-1 | 9 | umc109-umc1170 | 309.5-336.5 | 2940677-12687973 | 4.1145 | 13.51 | 11.5094 |
|  |  | qPH5-2 | 10 | phi054- umc2705 | 133.5-163.5 | 31183136-59002720 | 5.2032 | -10.9196 | 2.0847 |
|  |  | qPH5-3 | 10 | umc2221-umc1477 | 357.5-392.5 | 135966636-137333892 | 4.6775 | 6.8751 | 1.9042 |
| PH(RIL) | 2019Changchun | qPH6-1 | 3 | Marker2721323-Marker2723302 | 72.76-73.24 | 160148616 -161096774 | 5.567 | 6.489 | 6.809 |
|  | 2019Gongzhuling | qPH7-1 | 3 | Marker2715851-Marker2715956 | 71.54-72.03 | 157895072-157924932 | 5.050 | 6.075 | 6.699 |
|  | 2020Changchun | qPH8-1 | 10 | Marker7362705-Marker7353768 | 46.6-46.6 | 37215409- 41540766 | 4.505 | -10.605 | 14.035 |
|  | 2020Gongzhuling | qPH9-1 | 3 | Marker2366688-Marker2380844 | 28.68-28.92 | 93610111-101441466 | 5.090 | 6.105 | 8.547 |
| EH(F_2:3_) | 2016 Changchun | qEH1-1 | 2 | umc1422-umc1525 | 258.5-284.5 | 14128413-233654880 | 3.42 | 11.02 | 3.93 3.9269 11.0245 |
|  | 2017Changchun | qEH2-1 | 1 | umc1723a-bnlg1803 | 23.5-39.5 | 12448970-28638545 | 7.18 | 9.33 | 11.43 |
|  | 2017Gongzhuling | qEH3-1 | 3 | umc2265-umc1839 | 306.5-319.5 | 157012964-162111302 | 7.19 | 9.08 | 13.38 |
|  | 2018Changchun | qEH4-1 | 10 | umc1824c-umc1589 | 411.5- 426.5 | 95315410-110657182 | 8.24 | -11.57 | 16.21 |
|  | 2018Gongzhuling | qEH5-1 | 3 | bnlg1447-umc2258 | 0.00-10.5 | 9366940-10752207 | 3.29 | 5.61 | 4.12 |
|  |  | qEH5-2 | 3 | umc2265-umc1839 | 296.5-314.5 | 157012964-162111302 | 7.30 | 8.59 | 10.23 |
|  |  | qEH5-3 | 4 | umc2287-umc1371 | 32.5- 42.5 | 138472340-236999463 | 6.49 | 6.18 | 10.53 |
| EH(RIL)) | 2019Changchun | qEH6-1 | 1 | Marker53354-Marker57584 | 19.92-20.64 | 16559898-17802157 | 3.164 | 3.371 | 5.035 |
|  |  | qEH6-2 | 3 | Marker2721323-Marker2723302 | 72.76-73.24 | 160148616- 161096774 | 5.015 | 4.364 | 8.440 |
|  | 2019Gongzhuling | qEH7-1 | 1 | Marker45511-Marker47339 | 14.54-15.02 | 12642577-14705530 | 6.144 | 4.503 | 10.085 |
|  | 2020Changchun | qEH8-1 | 3 | Marker2709729-Marker2714212 | 70.58-70.82 | 156884455-157423737 | 4.439 | 4.953 | 8.866 |
|  | 2020Gongzhuling | qEH9-1 | 10 | Marker7708458-Marker7708492 | 83.87-84.35 | 136640675-136643297 | 4.073 | -3.968 | 6.215 |
|  |  | qEH9-2 | 3 | Marker2688319-Marker2691034 | 63.79-64.27 | 148507321-148640700 | 3.946 | 4.419 | 7.708 |
| LA(F_2:3_) | 2016 Changchun | qLA1-1 | 3 | umc2268-umc1641 | 376.5-385.5 | 184713010-230956846 | 3.531 | 2.735 | 0.759 |
|  |  | qLA1-2 | 7 | bnlg1200-phi057 | 869.5-894.5 | 11075377-17216464 | 2.693 | 4.04 | 4.853 |
|  |  | qLA1-3 | 9 | umc1893-umc109 | 246.5-269.5 | 2940677-18926278 | 4.304 | 4.454 | 3.392 |
|  |  | qLA1-4 | 9 | umc109-umc1170 | 281.5-317.5 | 2940677-12687973 | 3.88 | 3.958 | 3.074 |
|  |  | qLA1-5 | 10 | umc2705-umc1318 | 175.5-211.5 | 4246280-31183136 | 2.716 | 0.122 | 3.696 3.6963.696 |
|  | 2017Changchun | qLA2-1 | 3 | umc1773-umc2127 | 246.5-270.5 | 119806037-143262466 | 3.091 | 1.892 | 4.829 |
|  |  | qLA2-2 | 3 | umc2268- umc1641 | 376.5-385.5 | 184713010-230956846 | 13.898 | 5.905 | 24.226 |
|  | 2017Gongzhuling | qLA3-1 | 2 | umc1769- umc1555 | 148.5-182.5 | 24166809-213805344 | 2.660 | -0.300 | 4.742 |
|  |  | qLA3-2 | 4 | umc1317-bnlg1917 | 166.5-191.5 | 138472340-244755447 | 3.018 | 5.310 | 5.583 |
|  |  | qLA3-3 | 5 | umc2307-bnlg1711 | 313.5- 317 | 221714098-221195976 | 5.807 | 4.053 | 1.631 |
|  |  | qLA3-4 | 9 | umc109 -umc1170 | 320.5-349.5 | 2940677-12687973 | 3.492 | -5.399 | 5.324 |
|  | 2018Changchun | qLA4-1 | 3 | umc2268-umc1641 | 376.5-385.5 | 184713010-230956846 | 5.097 | 4.583 | 10.413 |
|  | 2018Gongzhuling | qLA5-1 | 2 | umc1525-umc1934 | 349.5-366.5 | 10606800-233654880 | 4.694 | -6.343 | 0.736 |
|  |  | qLA5-2 | 3 | umc2071- umc2048 | 159.5-186.5 | 1958218-234077205 | 5.044 | -6.771 | 0.734 |
|  |  | qLA5-3 | 5 | umc1766-umc1990 | 139.5-156.5 | 6175373-142583803 | 2.90 | 0.679 | 4.971 |
|  |  | qLA5-4 | 7 | phi328175-umc2092 | 188.5-233.5 | 118041132-168544322 | 4.743 | -5.80 | 0.795 |
|  |  | qLA5-5 | 7 | umc1865-umc1270 | 520.5-639.5 | 11773225-152372379 | 5.1541 | 6.617 | 0.662 |
|  |  | qLA5-6 | 7 | phi057-umc2160 | 943.5-962.5 | 10458921-11075377 | 4.4977 | 6.849 | 0.606 |
|  |  | qLA5-7 | 9 | umc1170-umc1867 | 397.5-477.5 | 4689770-12687973 | 3.838 | 6.254 | 0.656 |
| LA(RIL) | 2019Changchun | qLA6-1 | 5 | Marker4625126-Marker4625124 | 143.76-143.76 | 221518112-221518142 | 3.065 | 1.245 | 5.304 |
|  | 2019Gongzhuling | qLA7-1 | 1 | Marker911423-Marker923058 | 104.28-104.76 | 233396230-233865346 | 6.237 | 1.891 | 8.813 |
|  | 2020Changchun | qLA8-1 | 3 | Marker2790779-Marker2792880 | 106.13-106.13 | 186194870-186203649 | 4.602 | 1.722 | 8.679 |
|  | 2020Gongzhuling | qLA9-1 | 3 | Marker2790779-Marker279288 | 106.13-106.13 | 186194870-186203649 | 5.991 | 1.692 | 6.882 |
| IL(F_2:3_) | 2016 Changchun | qIL1-1 | 10 | umc1432-umc1367 | 75.5-90.5 | 5439443-26305370 | 3.659 | -2.107 | 0.793 |
|  | 2017Changchun | qIL2-1 | 1 | umc2226-umc1245 | 426.5-447.5 | 213709256-233337054 | 3.8860 | 0.709 | 2.920 |
|  |  | qIL2-2 | 4 | bnlg1265-umc1031 | 349.5-359 | 43883748-65618139 | 3.802 | -0.147 | 3.377 |
|  |  | qIL2-3 | 7 | umc1567-bnlg1305 | 362.5-381.5 | 134251695-134916785 | 5.961 | -0.263 | 6.433 |
|  | 2018Changchun | qIL4-1 | 2 | umc1227-bnlg1297 | 528.5-551.5 | 4666311- 4861458 | 4.551 | -0.906 | 3.802 |
|  |  | qIL4-2 | 10 | phi054-umc2705 | 130.5-135.5 | 31183136-59002720 | 26.608 | -2.812 | 30.748 |
|  |  | qIL4-3 | 10 | umc1824c-umc1589 | 418.5-432.5 | 95315410-110657182 | 13.989 | -2.260 | 19.545 |
|  | 2018Gongzhuling | qIL5-1 | 10 | phi054-umc2705 | 127.5-136.5 | 31183136-59002720 | 8.528 | -1.474 | 6.710 |
|  |  | qIL5-2 | 10 | umc1824c-umc1589 | 420.5-428.5 | 95315410-110657182 | 12.401 | -3.495 | 23.418 |
| IL(RIL) | 2019Changchun | qIL6-1 | 10 | Marker7617431-Marker7578581 | 71.42-71.42 | 95737789-107262109 | 5.530 | -0.401 | 9.861 |
|  | 2019Gongzhuling | qIL7-1 | 1 | Marker907046-Marker907566 | 101.38-101.62 | 229339491-229465187 | 2.181 | -0.317 | 3.701 |
|  | 2020Changchun | qIL8-1 | 1 | Marker1046781-Marker1046904 | 136.06-136.30 | 271938236-271946723 | 2.274 | -0.505 | 4.232 |
|  | 2020Gongzhuling | qIL9-1 | 2 | Marker1793927-Marker1835372 | 98.04-98.52 | 158045530-158044045 | 2.987 | -0.233 | 4.185 |
