# Supplementary material for: Maize plant architecture trait QTL mapping and candidate gene identification based on multiple environments and double populations
Source: BMC Plant Biol. 2022 Mar 11;22:110. doi: 10.1186/s12870-022-03470-7 (PMC8915473; doi:10.1186/s12870-022-03470-7)
Supplement: Supplementary file 9 — Additional file 9: Table S5. Names and data of phenotypic extreme value materials of plant architecture traits. [file 12870_2022_3470_MOESM9_ESM.doc]

|  |  | [material](../../../../C:/Program%20Files%20(x86)/Youdao/Dict/8.9.9.0/resultui/html/index.html#/javascript:;) [name](../../../../C:/Program%20Files%20(x86)/Youdao/Dict/8.9.9.0/resultui/html/index.html#/javascript:;) | 2019-Gongzhuling | 2019-Gongzhuling | 2020-Gongzhuling | 2020-Gongzhuling |
| --- | --- | --- | --- | --- | --- | --- |
| PH | low | FG143 | 164.2 | 182.4 | 178 | 172 |
|  |  | FG-197 | 136.4 | 144 | 137 | 130 |
|  |  | FG-201 | 132.4 | 146.6 | 155 | 170 |
|  |  | FG-177 | 159 | 173.8 | 165 | 149 |
|  |  | FG-43 | 148 | 162 | 175 | 180 |
|  | high | FG-2 | 200.4 | 225 | 230 | 210 |
|  |  | FG-7 | 218 | 233 | 242 | 215 |
|  |  | FG-99 | 211 | 236 | 228 | 205 |
|  |  | FG-104 | 253 | 245 | 215 | 223 |
|  |  | FG-127 | 212 | 232 | 254 | 242 |
|  |  | [material](../../../../C:/Program%20Files%20(x86)/Youdao/Dict/8.9.9.0/resultui/html/index.html#/javascript:;) [name](../../../../C:/Program%20Files%20(x86)/Youdao/Dict/8.9.9.0/resultui/html/index.html#/javascript:;) | 2019-Gongzhuling | 2019-Gongzhuling | 2020-Gongzhuling | 2020-Gongzhuling |
| EH | low | FG-143 | 58 | 63 | 75 | 60 |
|  |  | FG-197 | 51 | 47 | 55 | 50 |
|  |  | FG-201 | 56 | 54 | 55 | 35 |
|  |  | FG-177 | 66 | 73 | 80 | 55 |
|  |  | FG-43 | 85 | 60.4 | 66 | 55 |
|  | high | FG-2 | 83.4 | 74.8 | 103 | 80 |
|  |  | FG-7 | 96 | 82.6 | 125 | 93 |
|  |  | FG-99 | 99 | 89.8 | 110 | 90 |
|  |  | FG-104 | 137 | 100 | 125 | 100 |
|  |  | FG-127 | 89 | 93.4 | 130 | 110 |
|  |  | [material](../../../../C:/Program%20Files%20(x86)/Youdao/Dict/8.9.9.0/resultui/html/index.html#/javascript:;) [name](../../../../C:/Program%20Files%20(x86)/Youdao/Dict/8.9.9.0/resultui/html/index.html#/javascript:;) | 2019-Gongzhuling | 2019-Gongzhuling | 2020-Gongzhuling | 2020-Gongzhuling |
| LAE | small | FG-96 | 23 | 18 | 20 | 21.33 |
|  |  | FG-129 | 20 | 16.88 | 25 | 21.64 |
|  |  | FG-149 | 18.2 | 20.17 | 17 | 23.98 |
|  |  | FG-209 | 24.2 | 22.5 | 22.69 | 18.42 |
|  |  | FG-208 | 20.17 | 21.4 | 22.27 | 18.94 |
|  | big | FG-187 | 28.17 | 30.33 | 38.64 | 38.43 |
|  |  | FG-154 | 33.14 | 38.6 | 35 | 30.11 |
|  |  | FG-114 | 31.86 | 26.6 | 33.67 | 38.43 |
|  |  | FG-67 | 38.75 | 27 | 45.71 | 38.2 |
|  |  | FG-81 | 33.29 | 24 | 37.5 | 29.25 |
|  |  | [material](../../../../C:/Program%20Files%20(x86)/Youdao/Dict/8.9.9.0/resultui/html/index.html#/javascript:;) [name](../../../../C:/Program%20Files%20(x86)/Youdao/Dict/8.9.9.0/resultui/html/index.html#/javascript:;) | 2019-Gongzhuling | 2019-Gongzhuling | 2020-Gongzhuling | 2020-Gongzhuling |
| ILE | short | FG-41 | 10.44 | 10.63 | 10 | 10.83 |
|  |  | FG-119 | 9.33 | 10 | 10.77 | 10.45 |
|  |  | FG-194 | 10.25 | 9.93 | 12.08 | 9.43 |
|  |  | FG-197 | 10.78 | 10 | 9.73 | 9.55 |
|  |  | FG-201 | 8.22 | 10.2 | 11.09 | 9.33 |
|  | long | FG-162 | 14.56 | 15.5 | 13.08 | 13.58 |
|  |  | FG-124 | 11.33 | 15.25 | 14.5 | 13.42 |
|  |  | FG-205 | 14.4 | 14 | 14.36 | 13.92 |
|  |  | FG-200 | 14.38 | 14.89 | 13.31 | 12.07 |
|  |  | FG171 | 14.38 | 14.89 | 13.31 | 12.07 |

Table S5：Names and data of phenotypic extreme value materials of plant architecture traits.
