# Supplementary material for: Maize plant architecture trait QTL mapping and candidate gene identification based on multiple environments and double populations
Source: BMC Plant Biol. 2022 Mar 11;22:110. doi: 10.1186/s12870-022-03470-7 (PMC8915473; doi:10.1186/s12870-022-03470-7)
Supplement: Supplementary file 10 — Additional file 10: Table S6. List of primers used for the qRT-PCR assay of the key genes involved in plant architecture traits. [file 12870_2022_3470_MOESM10_ESM.docx]

**Table S6.**

| **Name of Genes** | **Primer Sequences** | **Use** |
| --- | --- | --- |
| Zm00001d043000 S  Zm00001d043000 AS | AGGGAGATGGATGGGAGAG  TCAATTCTCGCTCTGAGTTCG | qRT-PCR assay |
| Zm00001d042292 S  Zm00001d042292 AS | GAGCAAGGCAGGGAGAAA  GGCGACGATGTTGACGA | qRT-PCR assay |
| Zm00001d025008 S  Zm00001d025008 AS | GCAGTTGTGCTTGGTGATTATG  GTTGTCAGAAGAGACCCACTTG | qRT-PCR assay |
| ActinII1S  ActinII1AS | GCTGTTCTTTCACTTTATGCAAG  CGCTCGGCTGAGGTGGTGAAGGA | Reference gene |

List of primers used for the qRT-PCR assay of the key genes involved in plant architecture traits.
